# Nickel-catalyzed cross-coupling of 2-fluorobenzofurans with arylboronic acids via aromatic C–F bond activation

**DOI:** 10.3762/bjoc.21.8

**Published:** 2025-01-15

**Authors:** Takeshi Fujita, Haruna Yabuki, Ryutaro Morioka, Kohei Fuchibe, Junji Ichikawa

**Affiliations:** 1 The Institute for Solid State Physics, The University of Tokyo, 5-1-5 Kashiwanoha, Kashiwa, Chiba 277-8581, Japan; 2 Division of Chemistry, Faculty of Pure and Applied Sciences, University of Tsukuba, 1-1-1 Tennodai, Tsukuba, Ibaraki 305-8571, Japan; 3 Sagami Chemical Research Institute, 2743-1 Hayakawa, Ayase, Kanagawa 252-1193, Japan

**Keywords:** arylboronic acid, benzofuran, C–F bond activation, cross-coupling, nickel

## Abstract

2-Fluorobenzofurans underwent efficient nickel-catalyzed coupling with arylboronic acids through the activation of aromatic C–F bonds. This method allowed us to successfully synthesize a range of 2-arylbenzofurans with various substituents. The reaction, which proceeded under mild conditions, involved β-fluorine elimination from nickelacyclopropanes formed by the interaction of 2-fluorobenzofurans with zero-valent nickel species. This protocol facilitates orthogonal coupling reactions of aromatic C–F and C–Br bonds with arylboronic acids.

## Introduction

The metal-catalyzed activation of aromatic carbon–fluorine (C–F) bonds is widely recognized as a challenging task in synthetic organic chemistry owing to their high bond dissociation energy compared to other aromatic C–X (X = Cl, Br, I) bonds [1–7]. This activation is essential for the late-stage functionalization of stable C–F bonds in complex molecules with reactive functional groups, providing an orthogonal approach to complex molecule synthesis. Despite considerable efforts to develop various catalytic systems, the activation of aromatic C–F bonds often requires high temperatures [1–7]. Therefore, methods for activating aromatic C–F bonds at ambient temperature remain underdeveloped.

**Scheme 1:**
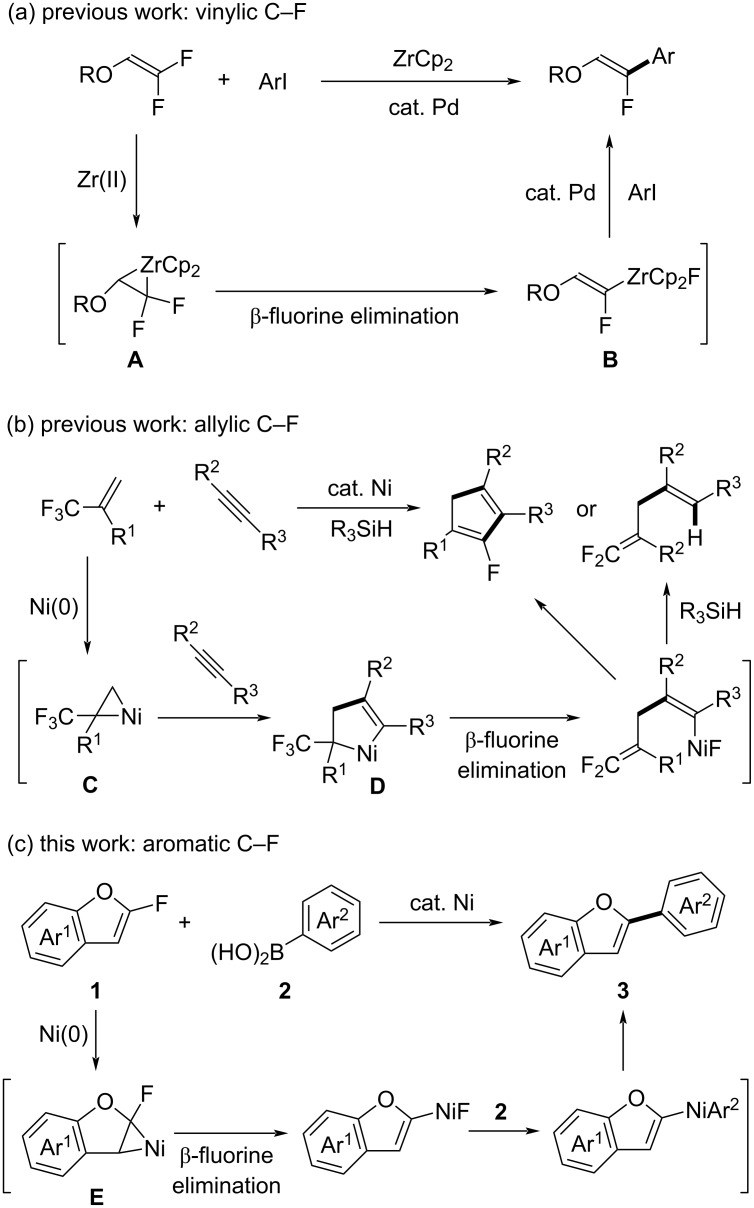
C–F bond activation through β-fluorine elimination via metalacyclopropanes.

We have developed efficient metal-mediated methods for activating (i) vinylic [8–13] and (ii) allylic C–F bonds [14–18] using β-fluorine elimination under mild conditions. In these studies, (i) we discovered zirconium-mediated β-fluorine elimination from zirconacyclopropanes **A**, which are generated by treating 1,1-difluoroethylenes with a zirconocene equivalent (ZrCp_2_, Scheme 1a) [8]. The resulting 1-fluorovinylzirconocenes **B** then undergo palladium-catalyzed coupling with aryl iodides to produce arylated fluoroethylenes. Additionally, (ii) we observed that electron-deficient 2-(trifluoromethyl)-1-alkenes strongly interact with electron-rich zero-valent nickel species to form nickelacyclopropanes **C** [15–17]. These intermediates enable C–F bond activation through the formation of nickelacyclopentenes **D** with alkynes, followed by β-fluorine elimination, leading to defluorinative coupling between these components (Scheme 1b).

Among aromatic fluorides, we have targeted 2-fluorobenzofurans **1** for C–F bond activation [19]. These compounds, which we prepared efficiently via 5-*endo*-*trig* cyclization of β,β-difluoro-*o*-hydroxystyrenes [20–21], possess a C–C double bond with an electron-deficient carbon atom owing to the nearby fluorine and oxygen atoms. We expected that 2-fluorobenzofurans **1** could form nickelacyclopropanes **E** upon treatment with zero-valent nickel species. Subsequent β-fluorine elimination from these intermediates **E** would facilitate the activation of aromatic C–F bonds (Scheme 1c). In this study, we demonstrate nickel-catalyzed defluorinative cross-coupling [22–37] of 2-fluorobenzofurans **1** with arylboronic acids **2** at ambient temperature, with nickelacyclopropanes **E** serving as crucial intermediates for the activation of aromatic C–F bonds.

## Results and Discussion

First, we explored optimal conditions for nickel-catalyzed defluorinative coupling using 2-fluoronaphtho[2,1-*b*]furan (**1b**) and *m*-tolylboronic acid (**2b**) as model substrates (Table 1). When **1b** was reacted with **2b** at 80 °C using Ni(cod)_2_ (10 mol %) as a catalyst, PCy_3_ (20 mol %) as a ligand, and K_2_CO_3_ (2.0 equiv) as a base, the desired arylated naphthofuran **3bb** was obtained in 75% yield (Table 1, entry 1). Reducing the reaction temperature improved the yield of **3bb**, reaching a quantitative yield when the reaction was performed at room temperature (Table 1, entry 3). Reducing the catalyst loading to 5 mol % slightly affected the yield of **3bb**, which was 90% (Table 1, entry 4). Next, we evaluated various additives with 5 mol % of Ni(cod)_2_ to stabilize regenerated zero-valent nickel species (Table 1, entries 5–8). While phosphine ligands such as triphenyl phosphite were ineffective (Table 1, entry 5), the inclusion of chelating dienes improved the yield of **3bb** (Table 1, entries 6–8). Among these, 5 mol % of 1,5-cyclooctadiene (cod) proved to be the most effective additive, affording **3bb** in 95% yield (Table 1, entry 8). Additionally, by reducing the equivalents of **2b** to 1.0 equiv and K_2_CO_3_ to 1.2 equiv, we achieved the highest yield of 98% for **3bb** (Table 1, entry 9).

**Table 1:** Screening of conditions for coupling of **1b** with **2b**.

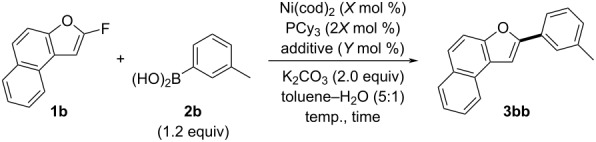						

Entry	*X*	Additive	*Y*	Temp.	Time (h)	**3bb** (%)

1	10	–	–	80 °C	24	75^a^
2	10	–	–	40 °C	72	91^a^
3	10	–	–	rt	72	quant.^a^
4	5	–	–	rt	28	90^b^
5	5	P(OPh)_3_	5	rt	58	12^b^
6	5	nbd^c^	5	rt	58	93^b^
7	5	chd^d^	5	rt	58	93^b^
8	5	cod^e^	5	rt	52	95^b^
9^f^	5	cod^e^	5	rt	14	98^b^

^a^Yield was determined by ^1^H NMR spectroscopy using CH_2_Br_2_ as an internal standard. ^b^Isolated yield. ^c^nbd = 2,5-norbornadiene. ^d^chd = 1,4-cyclohexadiene. ^e^cod = 1,5-cyclooctadiene. ^f^**2b** (1.0 equiv) and K_2_CO_3_ (1.2 equiv).

**Scheme 2:**
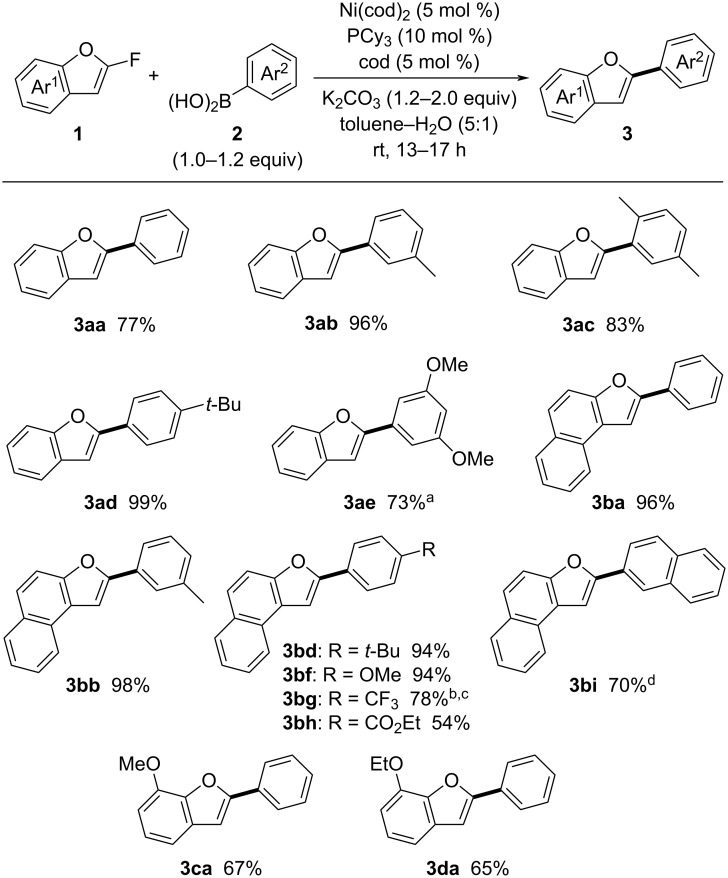
Synthesis of 2-arylbenzofurans **3** via the coupling of **1** with **2**. Isolated yields are given. ^a^Ni(cod)_2_ (10 mol %), PCy_3_ (20 mol %), and cod (10 mol %). ^b^Ni(cod)_2_ (20 mol %), PCy_3_ (40 mol %), and cod (20 mol %). ^c^K_3_PO_4_ (1.2 equiv) was used as a base. ^d^Toluene–MeOH–H_2_O (5:1:1) was used as a solvent.

Under the optimized conditions, we investigated the substrate scope using 2-fluorobenzofurans **1** and arylboronic acids **2** (Scheme 2). The coupling reaction was efficient with 2-fluorobenzofuran (**1a**) when reacted with phenylboronic acid (**2a**) as well as arylboronic acids containing electron-donating groups, such as a methyl group at the 3-position (**2b**), two methyl groups at the 2- and 5-positions (**2c**), and a *tert*-butyl group at the 4-position (**2d**). The reaction with 3,5-dimethoxyphenylboronic acid (**2e**), which has electron-withdrawing groups on the aromatic ring, also yielded a satisfactory result of 73%. Additionally, using 2-fluoronaphtho[2,1-*b*]furan (**1b**), the reaction with phenylboronic acid (**2a**) and arylboronic acids with a methyl group at the 3-position (**2b**) or a *tert*-butyl group at the 4-position (**2d**) also produced high yields (94–98%). For arylboronic acid **2f**, which has a methoxy group at the 4-position, the use of potassium phosphate as a base resulted in a 94% yield of **3bf**. For arylboronic acid **2g**, which features a strongly electron-withdrawing trifluoromethyl group, we optimized the coupling reaction using potassium phosphate as a base and increasing the nickel catalyst loading to 20 mol %, achieving a yield of 78% for the desired product **3bg**. When 2-naphthylboronic acid (**2i**) was employed, its solubility was enhanced using a mixed solvent system of toluene, methanol, and water, which effectively promoted the reaction and resulted in a 70% yield of **3bi**. Furthermore, when methoxy- and ethoxy-substituted benzofurans **1c** and **1d** were used, the corresponding coupling products **3ca** and **3da** were obtained with yields of 67% and 65%, respectively.

**Scheme 3:**
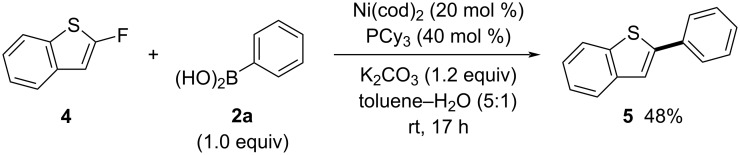
Synthesis of 2-phenylbenzothiophene (**5**).

Additionally, in the coupling reaction of 2-fluorobenzothiophene (**4**) with **2a**, increasing the amount of Ni(cod)_2_ to 20 mol % without adding extra cod yielded 48% of the desired product **5** (Scheme 3). This result indicates that the reaction is applicable to benzothiophenes as well as benzofurans.

**Scheme 4:**
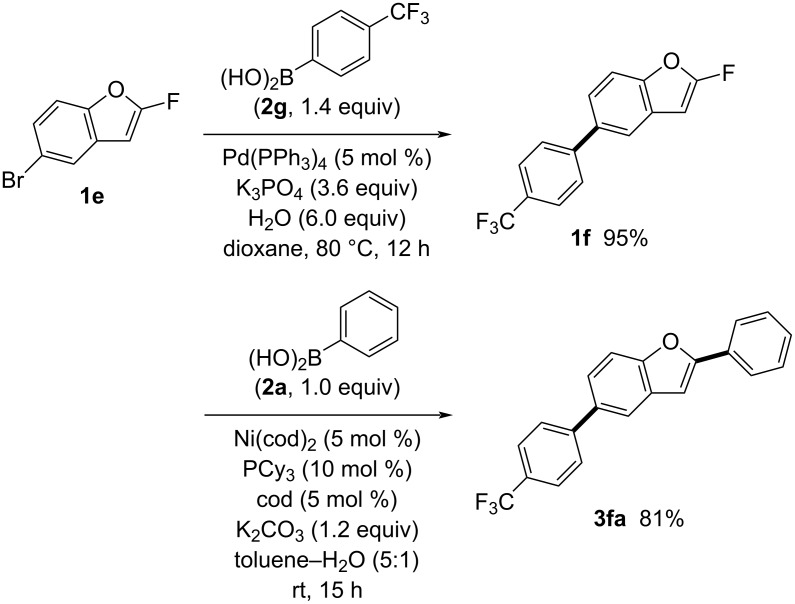
Orthogonal approach to 2,5-diarylbenzofuran **3fa**.

Moreover, we successfully introduced two distinct aryl groups onto a benzofuran ring through orthogonal coupling reactions, exploiting the reactivity difference between C–F and C–Br bonds (Scheme 4). Using a palladium catalyst, 5-bromo-2-fluorobenzofuran (**1e**) was coupled with [4-(trifluoromethyl)phenyl]boronic acid (**2g**). In this reaction, only the C–Br bond was transformed while the C–F bond remained intact, yielding 2-fluoro-5-[4-(trifluoromethyl)phenyl]benzofuran (**1f**) in 95% yield. Subsequently, nickel-catalyzed defluorinative arylation of **1f** with phenylboronic acid (**2a**) efficiently produced 2-phenyl-5-[4-(trifluoromethyl)phenyl]benzofuran (**3fa**) in 81% yield.

**Scheme 5:**
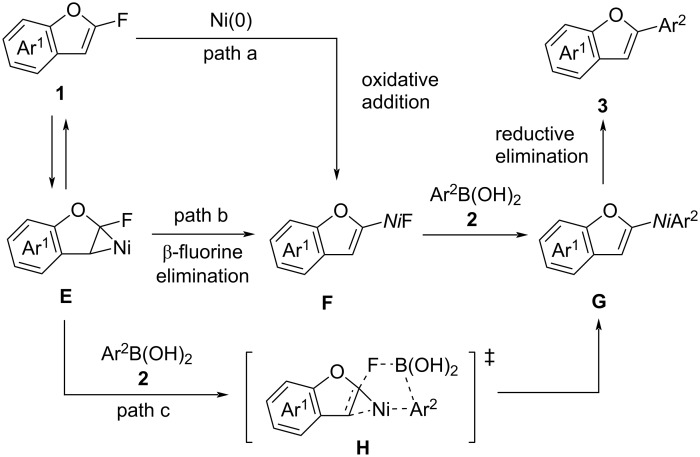
Possible mechanisms.

Next, we explored the mechanism of the coupling reactions between 2-fluorobenzofurans **1** and arylboronic acids **2**. Because these reactions proceed under mild conditions despite involving aromatic C–F bond activation [19], direct oxidative addition of C–F bonds is unlikely (Scheme 5, path a). Instead, the reactions are thought to proceed through a formal oxidative addition involving nickelacyclopropane intermediates **E** [15–17,38–39], which are generated from 2-fluorobenzofurans **1** and zero-valent nickel species (Scheme 5). Following β-fluorine elimination, this results in a formal oxidative addition to form benzofuranylnickel(II) fluorides **F**, which then undergo transmetallation with arylboronic acids **2** to produce intermediates **G** (Scheme 5, path b). Alternatively, a direct transition from **E** to **G** via transition state **H** is also possible (Scheme 5, path c). Ultimately, reductive elimination from **G** yields the coupling products **3**.

**Scheme 6:**
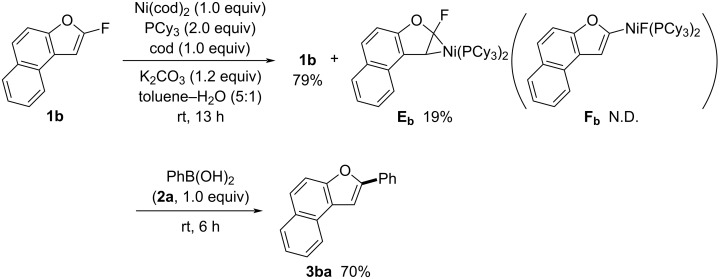
Formation of nickelacyclopropane **E_b_** in a stoichiometric reaction.

The following experiments were performed to elucidate the mechanism. Under the same conditions as the coupling reaction, stoichiometric amounts of Ni(cod)_2_, PCy_3_, and cod were treated with fluoronaphthofuran **1b** at room temperature for 13 h, excluding boronic acid **2a** (Scheme 6). The reaction was monitored using ^19^F and ^31^P NMR spectroscopy. The ^19^F NMR analysis showed that 79% of **1b** remained and revealed a new broad double doublet peak at 55.0 ppm (*J*_FP_ = 53, 42 Hz) relative to internal C_6_F_6_ (δ = 0.0 ppm). The ^31^P NMR spectrum depicted broad singlet peaks at 32.0–33.4 ppm and 38.6–40.5 ppm, appearing in a 1:1 ratio. These new peaks were attributed to nickelacyclopropane **E_b_**, which was formed in 19% yield. No peaks corresponding to benzofuranylnickel(II) fluoride **F_b_**, which would arise from the oxidative addition of **1b** to nickel(0), were detected [40]. High-resolution mass spectrometry (HRMS) analysis of the reaction mixture also supported the formation of **E_b_** (calcd, 804.4474; found, 804.4449). Additionally, 79% of **1b** remained, while the catalytic reaction between **1b** and **2a** was completed in 13 h, yielding **3ba** in 96% (Scheme 2). These findings suggest that nickelacyclopropanes **E** and 2-fluorobenzofurans **1** are in equilibrium (see Scheme 5). Consequently, in the absence of arylboronic acids **2**, the consumption of **1** was suppressed. Upon adding phenylboronic acid (**2a**, 1.0 equiv) to the above reaction mixture, the coupling proceeded, producing **3ba** in 70% yield, with neither complex **E_b_** nor **F_b_** observed (Scheme 6). These results suggest that nickelacyclopropanes **E** are initially formed and facilitate a formal oxidative addition. Notably, the absence of **F** in the reaction mixture indicates that fluorine elimination and transmetallation occur simultaneously between **E** and the arylboronic acids **2**, leading to the formation of **G** (Scheme 5, path c). The intermediates **G** then undergo reductive elimination to yield **3**.

To assess the impact of halogen substituents, we also examined reactions of 2-halogenated benzofurans **1a-X** (**1a-Cl**: X = Cl; **1a-Br**: X = Br; **1a-I**: X = I) with (3-methylphenyl)boronic acid (**2b**) (Table 2). Both 2-chlorobenzofuran (**1a-Cl**) and 2-bromobenzofuran (**1a-Br**) hardly yielded **3ab** under the optimized conditions for **1a** (Table 2, entries 2 and 3), while the reaction of 2-iodobenzofuran (**1a-I**) resulted in a much lower yield (32%) of 2-arylbenzofuran **3ab** (Table 2, entry 4) compared to that of **1a** (X = F, quant.). The strong interaction between fluorine and boron in **H** likely facilitates β-fluorine elimination and transmetallation. Thus, the considerably different result observed with **1a** is attributed to the distinct mechanistic aspects of the metalacyclopropanation/β-fluorine elimination sequence influenced by the fluorine substituent.

**Table 2:** Effect of halogen substituents.

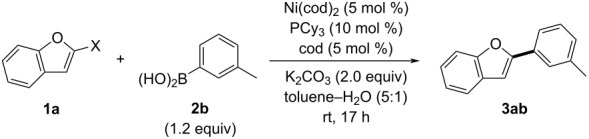			

Entry	**1a-X**	X	**3ab** (%)^a^

1	**1a**	F	quant.
2	**1a-Cl**	Cl	trace
3	**1a-Br**	Br	1
4	**1a-I**	I	32

^a^Yield was determined by ^1^H NMR spectroscopy using CH_2_Br_2_ as an internal standard.

## Conclusion

In summary, we have presented a nickel-catalyzed method for synthesizing 2-arylbenzofurans through aromatic C–F bond activation, with the formation of metallacyclopropanes as an essential step. This protocol allows for the late-stage transformation of C–F bonds, as demonstrated by the orthogonal activation of both aromatic C–F and C–Br bonds, thereby facilitating the synthesis of complex 2-arylbenzofurans. Given that natural and synthetic 2-arylbenzofurans often exhibit considerable biological activities and are important in pharmaceuticals and agrochemicals [41–47], we expect that this method will provide a novel and efficient approach for producing these valuable compounds.

## Experimental

**General:**^1^H NMR, ^13^C NMR, ^19^F NMR, and ^31^P NMR were recorded on a Bruker Avance 500 or a JEOL ECS-400 spectrometer. Chemical shift values are given in ppm relative to internal Me_4_Si (for ^1^H NMR: δ = 0.00 ppm), CDCl_3_ (for ^13^C NMR: δ = 77.0 ppm), C_6_F_6_ (for ^19^F NMR: δ = 0.0 ppm), and H_3_PO_4_ (for ^31^P NMR: δ = 0.0 ppm). IR spectra were recorded on a Horiba FT-730 spectrometer. Mass spectra were measured on a JEOL JMS-T100GCV or a JEOL JMS-T200GC spectrometer. All the reactions were conducted under argon or nitrogen.

**Materials:** Column chromatography was conducted on silica gel (Silica Gel 60 N, Kanto Chemical Co., Inc.). Toluene and *N*,*N*-dimethylformamide (DMF) were purified by a solvent-purification system (GlassContour) equipped with columns of activated alumina and supported-copper catalyst (Q-5) before use. 1,4-Dioxane and methanol were distilled from sodium, and stored over 4 Å molecular sieves. Unless otherwise noted, materials were obtained from commercial sources and used directly without further purifications.

**Typical procedure for coupling of 2-fluorobenzofurans 1 with arylboronic acids 2:** To the mixture of 2-fluoronaphtho[2,1-*b*]furan (**1b**, 56 mg, 0.30 mmol), (3-methylphenyl)boronic acid (**2b**, 41 mg, 0.30 mmol), Ni(cod)_2_ (4.2 mg, 0.015 mmol), PCy_3_ (8.2 mg, 0.029 mmol), 1,5-cyclooctadiene (1.8 μL, 0.015 mmol), and K_2_CO_3_ (50 mg, 0.36 mmol) were added toluene (3.0 mL) and H_2_O (0.6 mL). After stirring at room temperature for 13 h, the reaction mixture was diluted with H_2_O. Organic materials were extracted with diethyl ether three times. The combined extracts were washed with brine and dried over Na_2_SO_4_. After removal of the solvent under reduced pressure, the residue was purified by silica gel column chromatography (hexane/EtOAc = 10:1) to give **3bb** (76 mg, 98%) as a white solid. ^1^H NMR (500 MHz, CDCl_3_) δ 8.15 (d, *J* = 8.2 Hz, 1H), 7.93 (d, *J* = 8.2 Hz, 1H), 7.75–7.67 (m, 4H), 7.58 (ddd, *J* = 8.2, 6.9, 1.2 Hz, 1H), 7.49–7.46 (m, 2H), 7.35 (dd, *J* = 7.7, 7.6 Hz, 1H), 7.16 (d, *J* = 7.6 Hz, 1H), 2.44 (s, 3H); ^13^C NMR (126 MHz, CDCl_3_) δ 155.6, 152.3, 138.5, 130.5, 130.4, 129.1, 128.8, 128.7, 127.6, 126.2, 125.3, 125.1, 124.6, 124.5, 123.4, 121.9, 112.3, 100.3, 21.5; IR (KBr): 3051, 1606, 1487, 1387, 1280, 1255, 1163, 1053, 991, 935, 789, 690 cm^–1^; HREIMS *m*/*z*: [M]^+^ calcd for C_19_H_14_O, 258.1045; found, 258.1035.

## Supporting Information

File 1 Detailed experimental procedures and spectral data.

## Data Availability

All data that supports the findings of this study is available in the published article and/or the supporting information of this article.
