# Supplementary material for: Nickel-catalyzed cross-coupling of 2-fluorobenzofurans with arylboronic acids via aromatic C–F bond activation
Source: Beilstein J Org Chem. 2025 Jan 15;21:146–54. doi: 10.3762/bjoc.21.8 (PMC11744694; doi:10.3762/bjoc.21.8)
Supplement: File 1 — Detailed experimental procedures and spectral data. [file Beilstein_J_Org_Chem-21-146-s001.pdf]

BEILSTEIN JOURNAL OF ORGANIC CHEMISTRY

Supporting Information

for

Nickel-catalyzed cross-coupling of 2-fluorobenzofurans with arylboronic acids via aromatic C–F bond activation

Takeshi Fujita, Haruna Yabuki, Ryutaro Morioka, Kohei Fuchibe and Junji Ichikawa

Beilstein J. Org. Chem. **2025,** *21,* 146–154. doi:10.3762/bjoc.21.8

Detailed experimental procedures and spectral data

License and Terms: This is a supporting information file under the terms of the Creative Commons Attribution License (https://creativecommons.org/licenses/by/4.0). Please note that the reuse, redistribution and reproduction in particular requires that the author(s) and source are credited and that individual graphics may be subject to special legal provisions.
The license is subject to the *Beilstein Journal of Organic Chemistry* terms and conditions: (https://www.beilstein-journals.org/bjoc/terms)

1. General statement ······ S2

2. Preparation of 2-fluorobenzofurans ······ S2

3. Synthesis of 2-arylbenzofurans ······ S4

4. Orthogonal coupling reactions of aromatic C–F and C–Br bonds ······ S14

5. Formation of nickelacyclopropane E_b ······ S16

6. References ······ S17

7. ^{1}H, ^{13}C, and ^{19}F NMR charts ······ S18

8. NMR and HRMS charts of nickelacyclopropane E_b ······ S38

1. General statement

^{1}H NMR, ^{13}C NMR, ^{19}F NMR, and ^{31}P NMR were recorded on a Bruker Avance 500 or a JEOL ECS-400 spectrometer. Chemical shift values are given in ppm relative to internal Me_4Si (for ^{1}H NMR: δ = 0.00 ppm), $CDCl_3$ (for ^{13}C NMR: δ = 77.0 ppm), C_6F_6 (for ^{19}F NMR: δ = 0.0 ppm), and H_3PO_4 (for ^{31}P NMR: δ = 0.0 ppm). IR spectra were recorded on a Horiba FT-730 spectrometer. Mass spectra were measured on a JEOL JMS-T100GCV or a JEOL JMS-T200GC spectrometer.

Column chromatography was conducted on silica gel (Silica Gel 60 N, Kanto Chemical Co., Inc.). Toluene and *N*,*N*-dimethylformamide (DMF) were purified by a solvent-purification system (GlassContour) equipped with columns of activated alumina and supported-copper catalyst (Q-5) before use. 1,4-Dioxane and methanol were distilled from sodium, and stored over molecular sieves 4 Å. Unless otherwise noted, materials were obtained from commercial sources and used directly without further purifications.

2. Preparation of 2-fluorobenzofurans

2-Fluorobenzofuran (**1a**) [1], 2-fluoronaphtho[2,1-*b*]furan (**1b**) [1], 2-fluoro-7-methoxybenzofuran (**1c**) [1], 2-fluorobenzo[*b*]thiophene (**4**) [2], 5-bromo-2-fluorobenzofuran (**1e**) [1], 2-chlorobenzofuran (**1a-Cl**) [3], 2-bromobenzofuran (**1a-Br**) [4], and 2-iodobenzofuran (**1a-I**) [5] were prepared according to the literature procedures, and their spectral data showed good agreement with the literature data.

7-Ethoxy-2-benzofuran (1d)

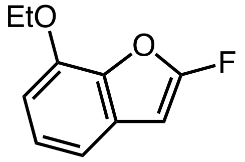

To a DMF (415 mL) solution of 2-(2,2-difluorovinyl)-6-ethoxyphenol (2.75 g, 13.7 mmol) was added DBU (2.46 mL, 16.5 mmol). After stirring at 100 °C for 2 h, water (250 mL) was added to the mixture. Organic materials were extracted with diethyl ether three times. The combined extracts were washed with brine and dried over Na_2SO_4. After removal of the solvent under reduced pressure, the residue was purified by silica gel column chromatography (pentane/diethyl ether = 10:1) to give **1d** (1.65 g, 67%) as a colorless liquid.

^{1}H NMR (500 MHz, $CDCl_3$): δ 7.13 (dd, J = 8.1, 7.8 Hz, 1H), 7.04 (dd, J = 7.8, 1.0 Hz, 1H), 6.77 (d, J = 8.1 Hz, 1H), 5.83 (d, J_{HF} = 6.7 Hz, 1H), 4.23 (q, J = 7.0 Hz, 2H), 1.50 (t, J = 7.0 Hz, 3H). ^{13}C NMR (126 MHz, $CDCl_3$): δ 160.2 (d, J_{CF} = 280 Hz), 144.1, 136.8, 129.6, 124.2, 112.9 (d, J_{CF} = 5 Hz), 107.3, 78.7 (d, J_{CF} = 13 Hz), 64.6, 14.9. ^{19}F NMR (470 MHz, $CDCl_3$): δ 49.5 (d, J_{FH} = 7 Hz, 1F). IR (neat): 3136, 3060, 2983, 2933, 1643, 1496, 1439, 1396, 1340, 1296, 1196, 1082, 1018, 978, 893, 783, 727, 658, 607, 555 cm^{-1}. HRMS (EI): *m/z* Calcd for $C_{10}H_9FO_2$ $[M]^+$: 180.0587; Found: 180.0592.

3. Synthesis of 2-Arylbenzofurans

2-(3-Methylphenyl)naphtho[2,1-*b*]furan (3bb)

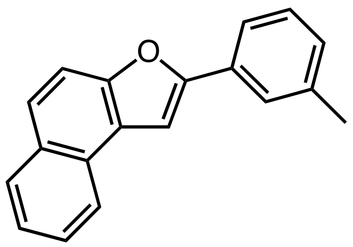

To the mixture of 2-fluoronaphtho[2,1-*b*]furan (**1b**, 56 mg, 0.30 mmol), (3-methylphenyl)boronic acid (**2b**, 41 mg, 0.30 mmol), Ni(cod)$_2$ (4.2 mg, 0.015 mmol), PCy$_3$ (8.2 mg, 0.029 mmol), 1,5-cyclooctadiene (1.8 μL, 0.015 mmol), and K_2CO_3 (50 mg, 0.36 mmol) were added toluene (3.0 mL) and H_2O (0.6 mL). After stirring at room temperature for 13 h, the reaction mixture was diluted with H_2O. Organic materials were extracted with diethyl ether three times. The combined extracts were washed with brine and dried over Na_2SO_4. After removal of the solvent under reduced pressure, the residue was purified by silica gel column chromatography (hexane/EtOAc = 10:1) to give **3bb** (76 mg, 98%) as a white solid.

^{1}H NMR (500 MHz, $CDCl_3$): δ 8.15 (d, *J* = 8.2 Hz, 1H), 7.93 (d, *J* = 8.2 Hz, 1H), 7.75–7.67 (m, 4H), 7.58 (ddd, *J* = 8.2, 6.9, 1.2 Hz, 1H), 7.49–7.46 (m, 2H), 7.35 (dd, *J* = 7.7, 7.6 Hz, 1H), 7.16 (d, *J* = 7.6 Hz, 1H), 2.44 (s, 3H). ^{13}C NMR (126 MHz, $CDCl_3$): δ 155.6, 152.3, 138.5, 130.5, 130.4, 129.1, 128.8, 128.7, 127.6, 126.2, 125.3, 125.1, 124.6, 124.5, 123.4, 121.9, 112.3, 100.3, 21.5. IR (KBr): 3051, 1606, 1487, 1387, 1280, 1255, 1163, 1053, 991, 935, 789, 690 cm^{-1}. HRMS (EI): *m*/*z* Calcd for $C_{19}H_{14}O$ [M]$^+$: 258.1045; Found: 258.1035.

2-Phenylbenzofuran (3aa)

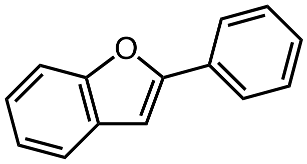

Compound **3aa** was synthesized by the method described for compound **3bb** using 2-fluorobenzofuran (**1a**, 28 mg, 0.20 mmol), phenylboronic acid (**2a**, 29 mg, 0.24 mmol), Ni(cod)$_2$ (2.8 mg, 0.010 mmol), PCy$_3$ (5.6 mg, 0.020 mmol), 1,5-cyclooctadiene (1.2 µL, 0.010 mmol), K_2CO_3 (55 mg, 0.40 mmol), toluene (2.0 mL), and H_2O (0.4 mL).

A white solid, 31 mg, 77% yield.

^{1}H NMR (500 MHz, CDCl$_3$): δ 7.85 (d, *J* = 7.8 Hz, 2H), 7.57 (d, *J* = 7.8 Hz, 1H), 7.51 (d, *J* = 7.8 Hz, 1H), 7.43 (dd, *J* = 7.8, 7.5 Hz, 2H), 7.33 (t, *J* = 7.5 Hz, 1H), 7.29–7.20 (m, 2H), 7.00 (s, 1H). ^{13}C NMR (126 MHz, CDCl$_3$): δ 155.9, 154.8, 130.4, 129.2, 128.8, 128.5, 124.9, 124.2, 122.9, 120.9, 111.1, 101.3.

Spectral data for this compound showed good agreement with literature data [6].

2-(3-Methylphenyl)benzofuran (3ab)

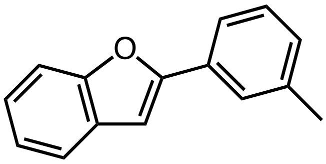

Compound **3ab** was synthesized by the method described for compound **3bb** using 2-fluorobenzofuran (**1a**, 28 mg, 0.20 mmol), (3-methylphenyl)boronic acid (**2b**, 33 mg, 0.24 mmol), Ni(cod)$_2$ (2.8 mg, 0.010 mmol), PCy$_3$ (5.6 mg, 0.020 mmol), 1,5-cyclooctadiene (1.2 µL, 0.010 mmol), K_2CO_3 (55 mg, 0.40 mmol), toluene (2.0 mL), and H_2O (0.4 mL).

A white solid, 41 mg, 96% yield.

^{1}H NMR (500 MHz, $CDCl_3$): δ 7.69 (s, 1H), 7.67 (d, *J* = 7.9 Hz, 1H), 7.57 (d, *J* = 7.6 Hz, 1H), 7.52 (d, *J* = 8.1 Hz, 1H), 7.33 (dd, *J* = 7.6, 7.6 Hz, 1H), 7.29–7.21 (m, 2H), 7.16 (d, *J* = 7.6 Hz, 1H), 7.00 (s, 1H), 2.42 (s, 3H). ^{13}C NMR (126 MHz, $CDCl_3$): δ 156.1, 154.8, 138.4, 130.3, 129.3, 129.2, 128.7, 125.5, 124.1, 122.9, 122.1, 120.8, 111.1, 101.2, 21.5.

Spectral data for this compound showed good agreement with literature data [7].

2-(2,5-Dimethylphenyl)benzofuran (3ac)

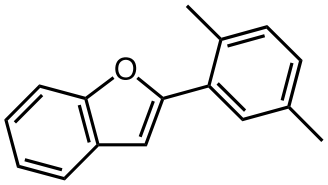

Compound **3ac** was synthesized by the method described for compound **3bb** using 2-fluorobenzofuran (**1a**, 27 mg, 0.20 mmol), (2,5-dimethylphenyl)boronic acid (**2c**, 36 mg, 0.24 mmol), $Ni(cod)_2$ (2.8 mg, 0.010 mmol), PCy_3 (5.6 mg, 0.020 mmol), 1,5-cyclooctadiene (1.2 μL, 0.010 mmol), K_2CO_3 (55 mg, 0.40 mmol), toluene (2.0 mL), and H_2O (0.4 mL).

A white solid, 37 mg, 83% yield.

^{1}H NMR (500 MHz, $CDCl_3$): δ 7.68 (s, 1H), 7.60 (d, *J* = 7.7 Hz, 1H), 7.52 (d, *J* = 8.1 Hz, 1H), 7.30–7.22 (m, 2H), 7.17 (d, *J* = 7.8 Hz, 1H), 7.09 (d, *J* = 7.8 Hz, 1H), 6.87 (s, 1H), 2.53 (s, 3H), 2.39 (s, 3H). ^{13}C NMR (126 MHz, $CDCl_3$): δ 155.8, 154.3, 135.5, 132.7, 131.2, 129.6, 129.3, 129.2, 128.6, 124.1, 122.7, 120.8, 111.0, 104.9, 21.4, 21.0.

Spectral data for this compound showed good agreement with literature data [6].

2-[4-(*tert*-Butyl)phenyl]benzofuran (3ad)

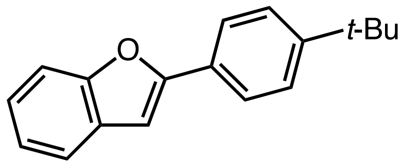

Compound **3ad** was synthesized by the method described for compound **3bb** using 2-fluorobenzofuran (**1a**, 29 mg, 0.21 mmol), [4-(*tert*-butyl)phenyl]boronic acid (**2d**, 43 mg, 0.24 mmol), Ni(cod)$_2$ (2.8 mg, 0.010 mmol), PCy$_3$ (5.6 mg, 0.020 mmol), 1,5-cyclooctadiene (1.2 μL, 0.010 mmol), K_2CO_3 (57 mg, 0.41 mmol), toluene (2.0 mL), and H_2O (0.4 mL).

A white solid, 53 mg, 99% yield.

^{1}H NMR (500 MHz, $CDCl_3$): δ 7.79 (d, *J* = 8.3 Hz, 2H), 7.55 (d, *J* = 7.6 Hz, 1H), 7.51 (d, *J* = 8.1 Hz, 1H), 7.46 (d, *J* = 8.3 Hz, 2H), 7.27–7.19 (m, 2H), 6.96 (s, 1H), 1.35 (s, 9H). ^{13}C NMR (126 MHz, $CDCl_3$): δ 156.1, 154.8, 151.8, 129.3, 127.7, 125.7, 124.7, 124.0, 122.8, 120.7, 111.1, 100.7, 34.7, 31.2.

Spectral data for this compound showed good agreement with literature data [8].

2-(3,5-Dimethoxyphenyl)benzofuran (3ae)

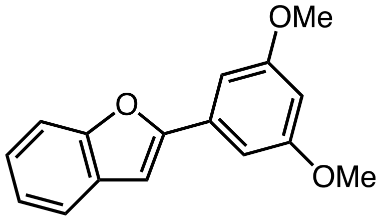

Compound **3ae** was synthesized by the method described for compound **3bb** using 2-fluorobenzofuran (**1a**, 28 mg, 0.20 mmol), (3,5-dimethoxyphenyl)boronic acid (**2e**, 44 mg, 0.24 mmol), Ni(cod)$_2$ (5.5 mg, 0.020 mmol), PCy$_3$ (11 mg, 0.040 mmol), 1,5-cyclooctadiene (2.5 μL, 0.020 mmol), K_2CO_3 (55 mg, 0.40 mmol), toluene (2.0 mL), and H_2O (0.4 mL).

A colorless oil, 38 mg, 73% yield.

^{1}H NMR (400 MHz, $CDCl_3$): δ 7.58 (d, *J* = 7.6 Hz, 1H) 7.52 (d, *J* = 8.1 Hz, 1H), 7.30–7.23 (m, 2H), 7.04–7.02 (m, 3H), 6.48 (t, *J* = 2.2 Hz, 1H), 3.88 (s, 6H). ^{13}C NMR (126 MHz, $CDCl_3$): δ 161.0, 155.6, 154.7, 132.1, 129.0, 124.3, 122.9, 120.9, 111.1, 102.9, 101.8, 101.0, 55.4.

Spectral data for this compound showed good agreement with literature data [8].

2-Phenylnaphtho[2,1-*b*]furan (3ba)

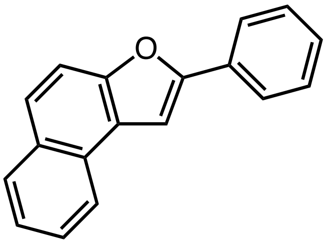

Compound **3ba** was synthesized by the method described for compound **3bb** using 2-fluoronaphtho[2,1-*b*]furan (**1b**, 56 mg, 0.30 mmol), phenylboronic acid (**2a**, 38 mg, 0.31 mmol), $Ni(cod)_2$ (4.2 mg, 0.015 mmol), PCy_3 (8.4 mg, 0.030 mmol), 1,5-cyclooctadiene (1.8 μL, 0.015 mmol), K_2CO_3 (50 mg, 0.36 mmol), toluene (3.0 mL), and H_2O (0.6 mL). A white solid, 71 mg, 96% yield.

^{1}H NMR (500 MHz, $CDCl_3$): δ 8.16 (d, *J* = 8.2 Hz, 1H) 7.95–7.91 (m, 3H), 7.72 (d, *J* = 9.0 Hz, 1H), 7.68 (d, *J* = 9.0 Hz, 1H), 7.59 (ddd, *J* = 8.1, 7.0, 1.2 Hz, 1H), 7.51–7.45 (m, 4H), 7.35 (t, *J* = 7.4 Hz, 1H). ^{13}C NMR (126 MHz, $CDCl_3$): δ 155.3, 152.3, 130.6, 130.4, 128.80, 128.76, 128.2, 127.6, 126.2, 125.1, 124.6, 124.52, 124.48, 123.4, 112.2, 100.4.

Spectral data for this compound showed good agreement with literature data [8].

2-[4-(*tert*-Butyl)phenyl]naphtho[2,1-*b*]furan (3bd)

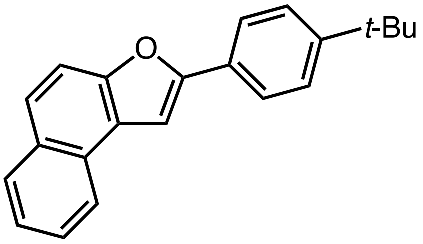

Compound **3bd** was synthesized by the method described for compound **3bb** using 2-fluoronaphtho[2,1-*b*]furan (**1b**, 55 mg, 0.30 mmol), [4-(*tert*-butyl)phenyl]boronic acid (**2d**, 54 mg, 0.30 mmol), Ni(cod)$_2$ (4.3 mg, 0.016 mmol), PCy$_3$ (8.2 mg, 0.029 mmol), 1,5-cyclooctadiene (1.8 µL, 0.015 mmol), K_2CO_3 (51 mg, 0.37 mmol), toluene (3.0 mL), and H_2O (0.6 mL).

A white solid, 84 mg, 94% yield.

^{1}H NMR (400 MHz, CDCl$_3$): δ 8.15 (d, *J* = 8.2 Hz, 1H), 7.93 (d, *J* = 8.1 Hz, 1H), 7.86–7.84 (m, 2H), 7.68–7.66 (m, 2H), 7.57–7.55 (m, 1H), 7.49–7.46 (m, 4H), 1.36 (s, 9H).

^{13}C NMR (100 MHz, CDCl$_3$): δ 155.6, 152.2, 151.5, 130.4, 128.8, 127.9, 127.6, 127.4, 126.1, 125.8, 124.8, 124.6, 124.5, 123.5, 112.3, 99.8, 34.7, 31.3.

Spectral data for this compound showed good agreement with literature data [9].

2-(4-Methoxyphenyl)naphtho[2,1-*b*]furan (3bf)

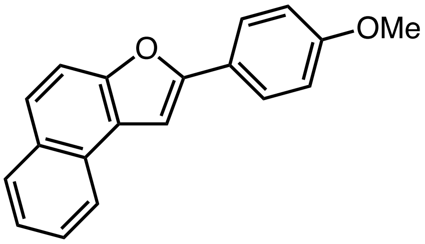

Compound **3bf** was synthesized by the method described for compound **3bb** using 2-fluoronaphtho[2,1-*b*]furan (**1b**, 39 mg, 0.21 mmol), (4-methoxyphenyl)boronic acid (**2f**, 38 mg, 0.25 mmol), Ni(cod)$_2$ (2.8 mg, 0.010 mmol), PCy$_3$ (5.6 mg, 0.020 mmol), 1,5-

cyclooctadiene (1.2 μL, 0.010 mmol), K_2CO_3 (55 mg, 0.40 mmol), toluene (2.0 mL), and H_2O (0.4 mL).

A white solid, 54 mg, 94% yield.

^{1}H NMR (400 MHz, $CDCl_3$): δ 8.14 (d, *J* = 8.2 Hz, 1H), 7.93 (d, *J* = 8.1 Hz, 1H), 7.84 (d, *J* = 8.8 Hz, 2H), 7.70–7.65 (m, 2H), 7.59–7.47 (m, 2H), 7.36 (s, 1H), 6.99 (d, *J* = 8.8 Hz, 2H), 3.85 (s, 3H). ^{13}C NMR (126 MHz, $CDCl_3$): δ 159.8, 155.5, 152.0, 130.4, 128.7, 127.5, 126.12, 126.06, 124.7, 124.5, 124.4, 123.52, 123.45, 114.3, 112.2, 98.8, 55.3.

Spectral data for this compound showed good agreement with literature data [10].

2-[4-(Trifluoromethyl)phenyl]naphtho[2,1-*b*]furan (3bg)

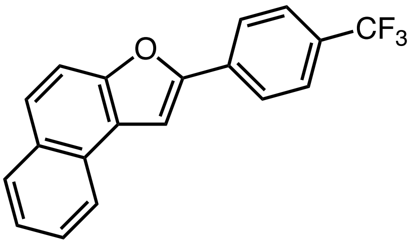

Compound **3bg** was synthesized by the method described for compound **3bb** using 2-fluoronaphtho[2,1-*b*]furan (**1b**, 56 mg, 0.30 mmol), [4-(trifluoromethyl)phenyl]boronic acid (**2g**, 57 mg, 0.30 mmol), Ni(cod)$_2$ (17 mg, 0.060 mmol), PCy_3 (34 mg, 0.12 mmol), 1,5-cyclooctadiene (7.4 μL, 0.060 mmol), K_3PO_4 (76 mg, 0.36 mmol), toluene (3.0 mL), and H_2O (0.6 mL).

A white solid, 74 mg, 78% yield.

^{1}H NMR (500 MHz, $CDCl_3$): δ 8.17 (d, *J* = 8.0 Hz, 1H), 8.01 (d, *J* = 8.3 Hz, 2H), 7.96 (d, *J* = 8.2 Hz, 1H), 7.78–7.69 (m, 4H), 7.63–7.61 (m, 2H), 7.52 (dd, *J* = 8.0, 7.1 Hz, 1H). ^{13}C NMR (100 MHz, $CDCl_3$): δ 153.6, 152.8, 133.8, 130.5, 129.8 (q, J_{CF} = 32 Hz), 128.9, 127.6, 126.6, 126.2, 125.9 (q, J_{CF} = 4 Hz), 124.9, 124.6, 124.3, 124.1 (q, J_{CF} = 271 Hz), 123.4, 112.3, 102.4. ^{19}F NMR (470 MHz, $CDCl_3$): δ 100.3 (s). IR (KBr): 3066,

2929, 1616, 1412, 1325, 1169, 1128, 1072, 1012, 922, 841, 802, 750, 675, 594 cm^{-1}. HRMS (EI): *m*/*z* Calcd for $C_{19}H_{11}F_3O$ $[M]^+$: 312.0762; Found: 312.0770.

Ethyl 4-(naphtho[2,1-*b*]furan-2-yl)benzoate (3bh)

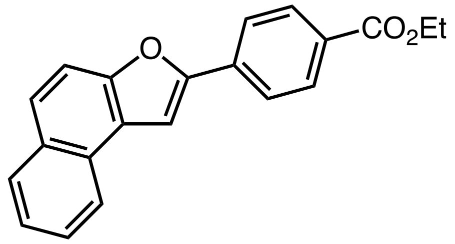

Compound **3bh** was synthesized by the method described for compound **3bb** using 2-fluoronaphtho[2,1-*b*]furan (**1b**, 38 mg, 0.20 mmol), [4-(ethoxycarbonyl)phenyl]boronic acid (**2h**, 47 mg, 0.24 mmol), Ni(cod)$_2$ (2.9 mg, 0.011 mmol), PCy_3 (5.7 mg, 0.020 mmol), 1,5-cyclooctadiene (1.2 µL, 0.010 mmol), K_2CO_3 (57 mg, 0.41 mmol), toluene (2.0 mL), and H_2O (0.4 mL).

A white solid, 35 mg, 54% yield.

^{1}H NMR (400 MHz, $CDCl_3$): δ 8.19–8.13 (m, 3H), 7.99–7.95 (m, 3H), 7.77 (d, *J* = 9.4 Hz, 1H), 7.70 (d, *J* = 9.4 Hz, 1H), 7.65 (s, 1H), 7.62 (ddd, *J* = 8.4, 6.8, 0.8 Hz, 1H), 7.51 (ddd, *J* = 8.2, 6.8, 1.4 Hz, 1H). ^{13}C NMR (100 MHz, $CDCl_3$): δ 166.2, 154.2, 152.9, 134.5, 130.5, 130.2, 129.8, 128.9, 127.6, 126.6, 126.1, 124.8, 124.4, 124.3, 123.4, 112.3, 102.5, 61.1, 14.4. IR (KBr): 3057, 2981, 1712, 1608, 1410, 1367, 1279, 1178, 1105, 1022, 989, 856, 810, 768, 690 cm^{-1}. HRMS (FD): *m*/*z* Calcd for $C_{21}H_{16}O_3$ $[M]^+$: 316.1099; Found: 316.1105.

2-(Naphthalen-2-yl)naphtho[2,1-*b*]furan (3bi)

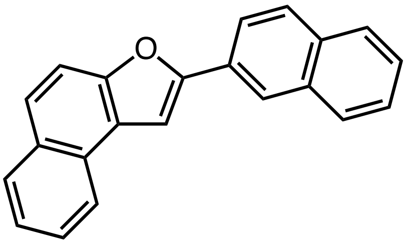

Compound **3bi** was synthesized by the method described for compound **3bb** using 2-fluoronaphtho[2,1-*b*]furan (**1b**, 56 mg, 0.30 mmol), 2-naphthylboronic acid (**2i**, 51 mg, 0.30 mmol), Ni(cod)$_2$ (4.1 mg, 0.015 mmol), PCy$_3$ (8.5 mg, 0.030 mmol), 1,5-cyclooctadiene (1.8 μL, 0.015 mmol), K_2CO_3 (50 mg, 0.36 mmol), toluene (3.0 mL), methanol (0.6 mL), and H_2O (0.6 mL).

A white solid, 62 mg, 70% yield.

^{1}H NMR (500 MHz, CDCl$_3$): δ 8.42 (s, 1H), 8.21 (d, *J* = 8.2 Hz, 1H), 8.01–7.92 (m, 4H), 7.86 (d, *J* = 8.0 Hz, 1H), 7.76 (d, *J* = 9.0 Hz, 1H), 7.74 (d, *J* = 9.0 Hz, 1H), 7.65 (s, 1H), 7.62 (ddd, *J* = 8.1, 6.9, 1.2 Hz, 1H), 7.55–7.51 (m, 3H). ^{13}C NMR (126 MHz, CDCl$_3$): δ 155.4, 152.5, 133.5, 133.1, 130.5, 128.8, 128.6, 128.4, 127.9, 127.8, 127.6, 126.7, 126.4, 126.3, 125.4, 124.63, 124.61, 123.5, 123.4, 122.7, 112.3, 101.1.

Spectral data for this compound showed good agreement with literature data [11].

7-Methoxy-2-phenylbenzofuran (3ca)

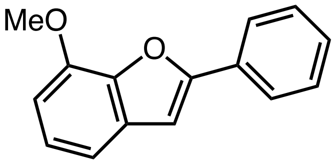

Compound **3ca** was synthesized by the method described for compound **3bb** using 2-fluoro-7-methoxybenzofuran (**1c**, 56 mg, 0.33 mmol), phenylboronic acid (**2a**, 37 mg, 0.31 mmol), Ni(cod)$_2$ (4.1 mg, 0.015 mmol), PCy$_3$ (8.1 mg, 0.029 mmol), 1,5-

cyclooctadiene (1.8 μL, 0.015 mmol), K_2CO_3 (50 mg, 0.36 mmol), toluene (3.0 mL), and H_2O (0.6 mL).

A white solid, 46 mg, 67% yield.

^{1}H NMR (500 MHz, $CDCl_3$): δ 7.89 (d, *J* = 7.4 Hz, 2H), 7.44 (dd, *J* = 7.9, 7.4 Hz, 2H), 7.36–7.33 (m, 1H), 7.19–7.13 (m, 2H), 7.01 (s, 1H), 6.80 (d, *J* = 6.7 Hz, 1H), 4.05 (s, 3H). ^{13}C NMR (126 MHz, $CDCl_3$): δ 156.0, 145.3, 144.1, 130.9, 130.3, 128.7, 128.5, 125.0, 123.6, 113.3, 106.6, 101.6, 56.1.

Spectral data for this compound showed good agreement with literature data [12].

7-Ethoxy-2-phenylbenzofuran (3da)

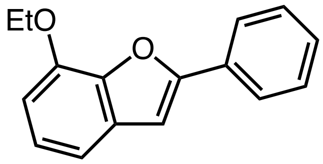

Compound **3da** was synthesized by the method described for compound **3bb** using 7-ethoxy-2-fluorobenzofuran (**1d**, 54 mg, 0.30 mmol), phenylboronic acid (**2a**, 37 mg, 0.31 mmol), Ni(cod)$_2$ (4.1 mg, 0.015 mmol), PCy$_3$ (8.1 mg, 0.029 mmol), 1,5-cyclooctadiene (1.8 μL, 0.015 mmol), K_2CO_3 (50 mg, 0.36 mmol), toluene (3.0 mL), and H_2O (0.6 mL).

A colorless oil, 46 mg, 65% yield.

^{1}H NMR (500 MHz, $CDCl_3$): δ 7.89 (d, *J* = 8.0 Hz, 2H), 7.44 (dd, *J* = 8.0, 7.5 Hz, 2H), 7.34 (t, *J* = 7.5 Hz, 1H), 7.17 (d, *J* = 7.6 Hz, 1H), 7.12 (dd, *J* = 7.8, 7.6 Hz, 1H), 7.00 (s, 1H), 6.80 (d, *J* = 7.8 Hz, 1H), 4.32 (q, *J* = 7.0 Hz, 2H), 1.54 (t, *J* = 7.0 Hz, 3H). ^{13}C NMR (126 MHz, $CDCl_3$): δ 155.9, 144.5, 144.3, 131.0, 130.4, 128.7, 128.5, 125.0, 123.5, 113.2, 108.1, 101.6, 64.7, 15.0.

Spectral data for this compound showed good agreement with literature data [12].

2-Phenylbenzo[*b*]thiophene (5)

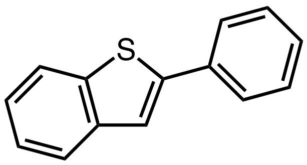

Compound **5** was synthesized by the method described for compound **3bb** using 2-fluorobenzo[*b*]thiophene (**4**, 45 mg, 0.29 mmol), phenylboronic acid (**2a**, 37 mg, 0.30 mmol), Ni(cod)$_2$ (17 mg, 0.060 mmol), PCy$_3$ (34 mg, 0.12 mmol), K_2CO_3 (50 mg, 0.36 mmol), toluene (3.0 mL), and H_2O (0.6 mL).

A white solid, 29 mg, 48% yield.

^{1}H NMR (500 MHz, $CDCl_3$): δ 7.82 (d, *J* = 7.8 Hz, 1H), 7.77 (d, *J* = 7.7 Hz, 1H), 7.71 (d, *J* = 7.5 Hz, 2H), 7.54 (s, 1H), 7.42 (dd, *J* = 7.9, 7.5 Hz, 2H), 7.36–7.29 (m, 3H). ^{13}C NMR (126 MHz, $CDCl_3$): δ 144.2, 140.7, 139.5, 134.3, 128.9, 128.2, 126.5, 124.5, 124.3, 123.5, 122.2, 119.4.

Spectral data for this compound showed good agreement with literature data [13].

4. Orthogonal coupling reactions of aromatic C–F and C–Br bonds

2-Fluoro-5-[4-(trifluoromethyl)phenyl]benzofuran (1f)

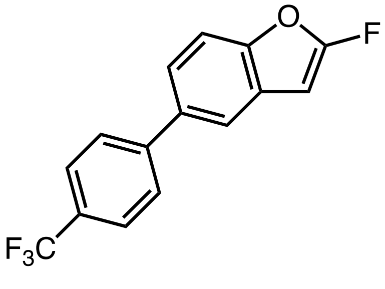

To the mixture of 5-bromo-2-fluorobenzofuran (**1e**, 357 mg, 1.66 mmol), [4-(trifluoromethyl)phenyl)]boronic acid (**2g**, 453 mg, 2.39 mmol), Pd(PPh$_3$)$_4$ (92 mg, 0.080 mmol), K_3PO_4 (1.27 g, 5.98 mmol), and H_2O (0.18 mL, 10 mmol) was added 1,4-dioxane (5.3 mL). After stirring at 80 °C for 12 h, the reaction mixture was diluted with

H_2O. Organic materials were extracted with dichloromethane three times. The combined extracts were washed with brine and dried over Na_2SO_4. After removal of the solvent under reduced pressure, the residue was purified by silica gel column chromatography (hexane) to give **1f** (444 mg, 95%) as a white solid.

^{1}H NMR (500 MHz, $CDCl_3$): δ 7.73–7.65 (m, 5H), 7.47–7.43 (m, 2H), 5.91 (d, J_{HF} = 6.6 Hz, 1H). ^{13}C NMR (126 MHz, $CDCl_3$): δ 160.9 (d, J_{CF} = 281 Hz), 147.7, 144.9, 135.9, 129.2 (q, J_{CF} = 32 Hz), 128.6 (d, J_{CF} = 3 Hz), 127.6, 125.7 (q, J_{CF} = 4 Hz), 124.3 (q, J_{CF} = 272 Hz), 123.0 (d, J_{CF} = 4 Hz), 119.5 (d, J_{CF} = 6 Hz), 111.3, 78.6 (d, J_{CF} = 14 Hz). ^{19}F NMR (376 MHz, $CDCl_3$): δ 100.5 (s, 3F), 52.3 (d, J_{FH} = 7 Hz, 1F). IR (KBr): 3149, 1637, 1468, 1323, 1165, 1124, 1068, 976, 843, 810, 777, 671 cm^{-1}. HRMS (EI): *m/z* Calcd for $C_{15}H_8F_4O$ $[M]^+$: 280.0511; Found: 280.0522.

2-Phenyl-5-[4-(trifluoromethyl)phenyl]benzofuran (3fa)

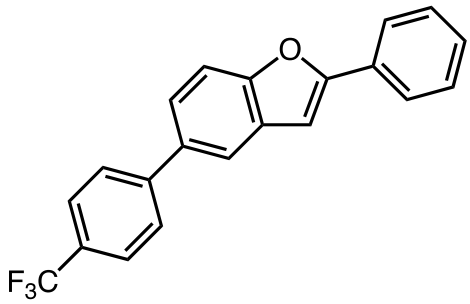

Compound **3fa** was synthesized by the method described for compound **3bb** using 2-fluoro-5-[4-(trifluoromethyl)phenyl]benzofuran (**1f**, 85 mg, 0.30 mmol), phenylboronic acid (**2a**, 38 mg, 0.31 mmol), Ni(cod)$_2$ (4.2 mg, 0.015 mmol), PCy_3 (8.2 mg, 0.029 mmol), 1,5-cyclooctadiene (1.8 μL, 0.015 mmol), K_2CO_3 (50 mg, 0.36 mmol), toluene (3.0 mL), and H_2O (0.6 mL).

A white solid, 83 mg, 81% yield.

^{1}H NMR (400 MHz, $CDCl_3$): δ 7.89 (d, J = 6.8 Hz, 2H), 7.78 (d, J = 1.6 Hz, 1H), 7.75–7.69 (m, 4H), 7.60 (d, J = 8.8 Hz, 1H), 7.52–7.45 (m, 3H), 7.38 (t, J = 7.4 Hz, 1H), 7.08

(s, 1H). ^{13}C NMR (100 MHz, $CDCl_3$): δ 157.0, 154.9, 145.2, 135.1, 130.2, 129.9, 129.0 (q, J_{CF} = 32 Hz), 128.85, 128.85, 127.6, 125.7 (q, J_{CF} = 4 Hz), 125.0, 124.5 (q, J_{CF} = 270 Hz), 123.9, 119.6, 111.5, 101.3. ^{19}F NMR (376 MHz, $CDCl_3$): δ 99.4 (s).

Spectral data for this compound showed good agreement with literature data [14].

5. Formation of nickelacyclopropane E_b

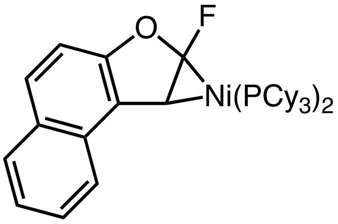

To the mixture of 2-fluoronaphtho[2,1-*b*]furan (**1b**, 18 mg, 0.098 mmol), $Ni(cod)_2$ (28 mg, 0.10 mmol), PCy_3 (56 mg, 0.20 mmol), 1,5-cyclooctadiene (12 µL, 0.098 mmol), and K_2CO_3 (17 mg, 0.12 mmol) were added toluene (1.0 mL) and H_2O (0.2 mL). After stirring at room temperature for 13 h, the formation of nickelacyclopropane $\mathbf{E_b}$ was confirmed by ^{19}F and ^{31}P NMR measurement and HRMS analysis.

^{19}F NMR (470 MHz, $CDCl_3$): δ 55.0 (br dd, J_{FP} = 53, 42 Hz). ^{31}P NMR (202 MHz, $CDCl_3$): δ 40.5–38.6 (br s, 1P), 33.4–32.0 (br s, 1P). HRMS (FD): *m*/*z* Calcd for $C_{48}H_{73}FNiOP_2$ $[M]^+$: 804.4474; Found: 804.4449.

6. References

1. Morioka, R.; Fujita, T.; Ichikawa, J. *Helv. Chim. Acta* **2020**, *103*, e2000159.
2. Hofsäß, R.; Rombach, D.; Wagenknecht, H.-A. *Synlett* **2017**, *28*, 1422–1426.
3. Liégault, B.; Petrov, I.; Gorelsky, S. I.; Fagnou, K. *J. Org. Chem.* **2010**, *75*, 1047–1060.
4. Chen, W.; Zhang, Y.; Zhang, L.; Wang, M.; Wang, L. *Chem. Commun.* **2011**, *47*, 10476–10478.
5. Tong, Z.; Garry, O. L.; Smith, P. J.; Jiang, Y.; Mansfield, S. J.; Anderson, E. A. *Org. Lett.* **2021**, *23*, 4888–4892.
6. Dwight, T. A.; Rue, N. R.; Charyk, D.; Josselyn, R.; DeBoef, B. *Org. Lett.* **2007**, *9*, 3137–3139.
7. Csékei, M.; Novák, Z.; Kotschy, A. *Tetrahedron* **2008**, *64*, 8992–8996.
8. Moure, M. J.; SanMartin, R.; Domínguez, E. *Adv. Synth. Catal.* **2014**, *356*, 2070–2080.
9. Liu, L.; Li, X.; Dong, J.; Zhou, Y.; Yin, S.-F. *Org. Lett.* **2016**, *18*, 3138–3141.
10. Pan, C.; Yu, J.; Zhou, Y.; Wang, Z.; Zhou, M.-M. *Synlett* **2006**, 1657–1662.
11. Gao, H.; Xu, Q.-L.; Keene, C.; Kürti, L. *Chem. Eur. J.* **2014**, *20*, 8883–8887.
12. Liu, J.; Chen, W.; Ji, Y.; Wang, L. *Adv. Synth. Catal.* **2012**, *354*, 1585–1592.
13. Kashiki, T.; Shinamura, S.; Kohara, M.; Miyazaki, E.; Takimiya, K.; Ikeda, M.; Kuwabara, H. *Org. Lett.* **2009**, *11*, 2473–2475.
14. Yamaguchi, M.; Katsumata, H.; Manabe, K. *J. Org. Chem.* **2013**, *78*, 9270–9281.

7. ^{1}H, ^{13}C, and ^{19}F NMR charts

2-Phenylbenzofuran (3aa)

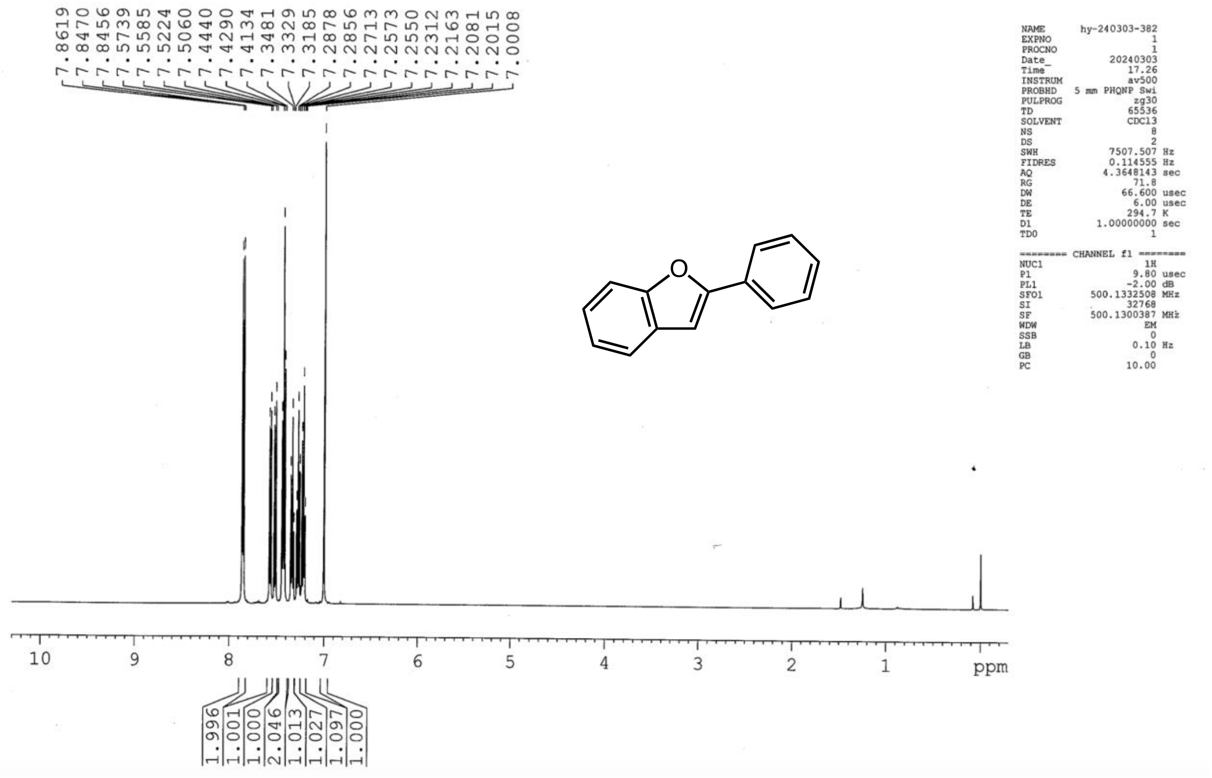

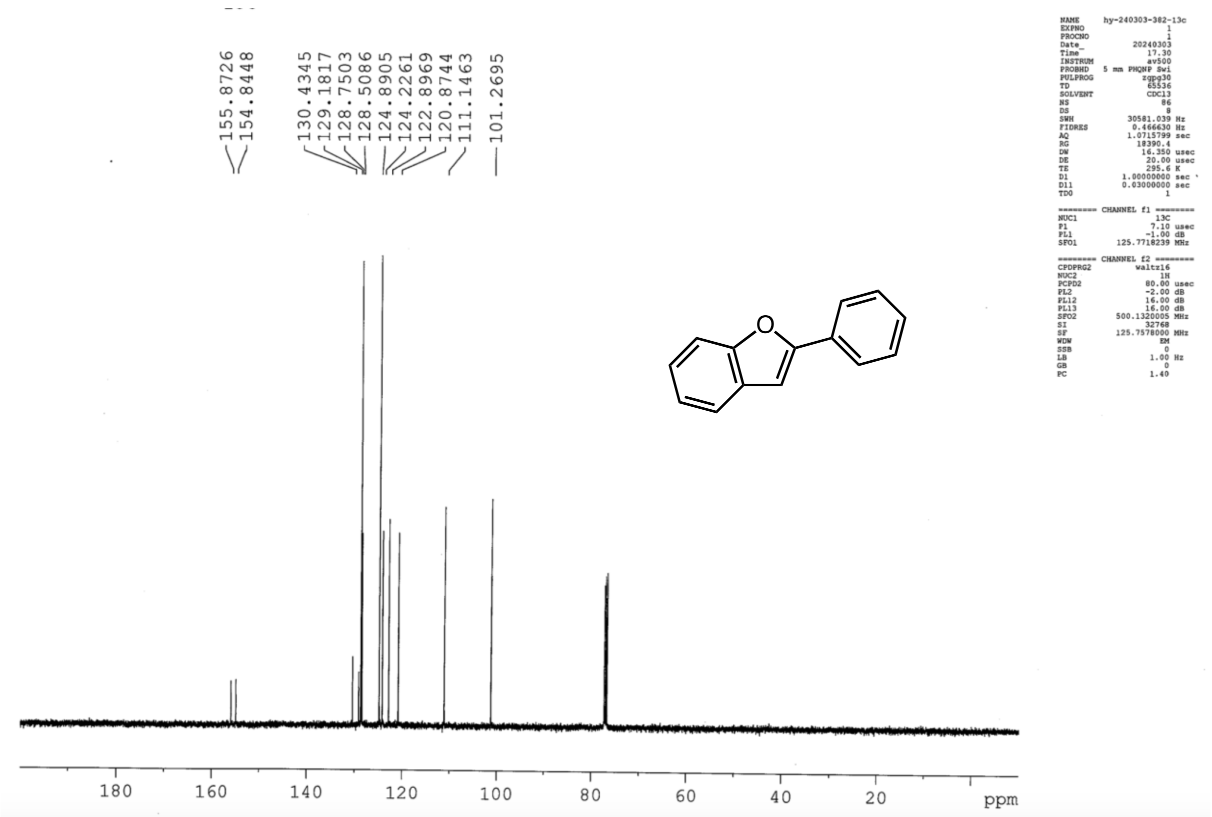

2-(3-Methylphenyl)benzofuran (3ab)

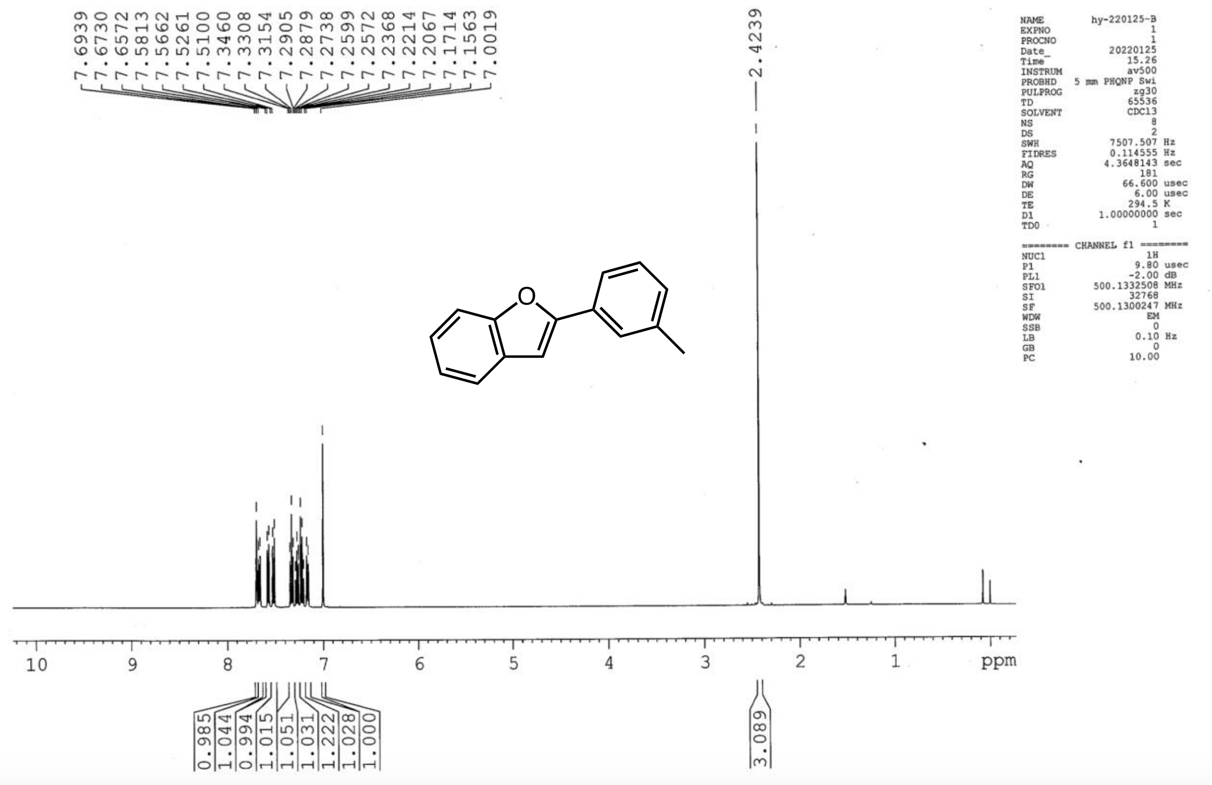

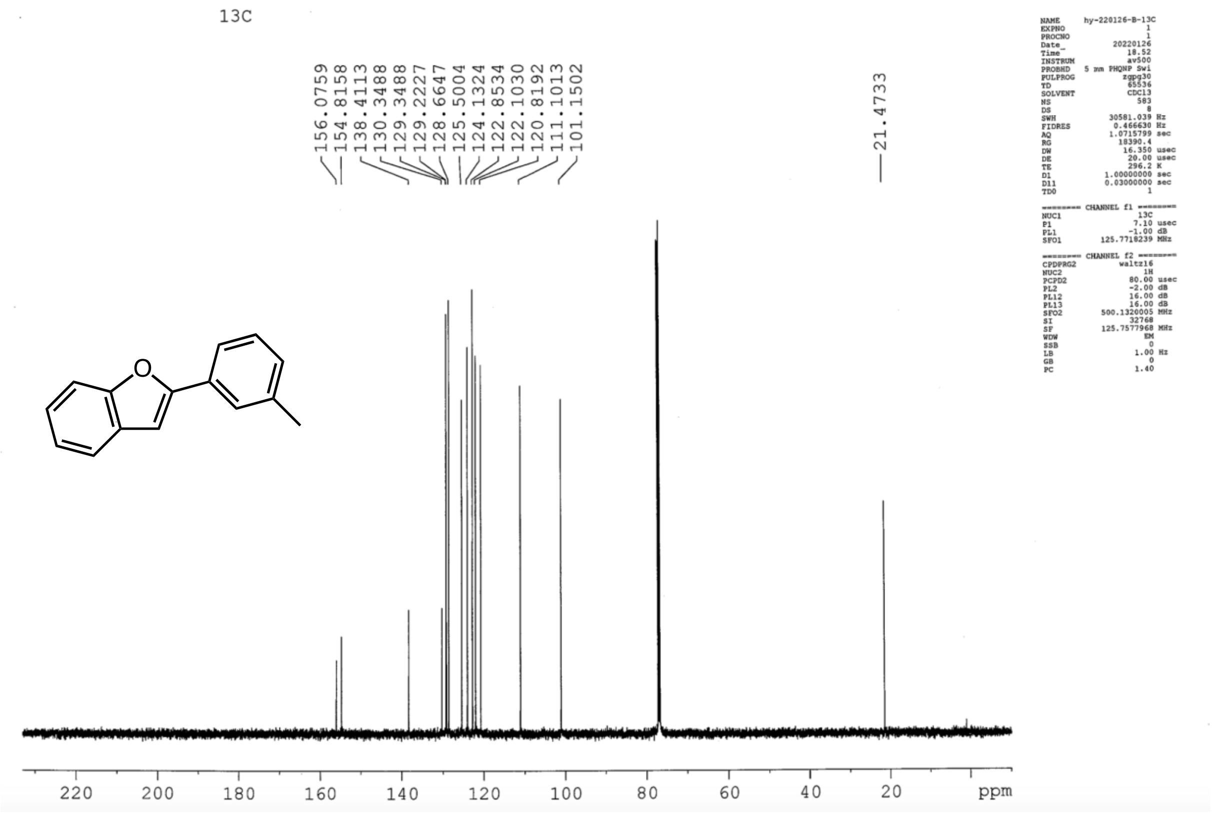

2-(2,5-Dimethylphenyl)benzofuran (3ac)

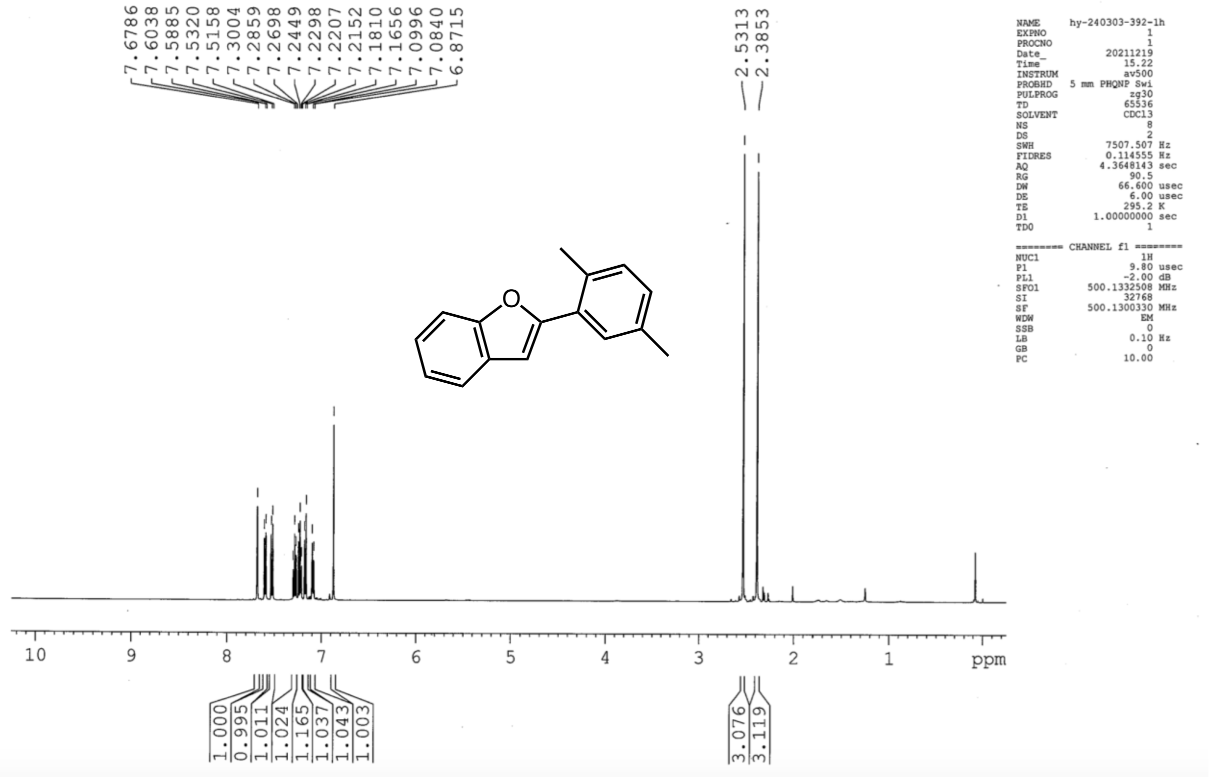

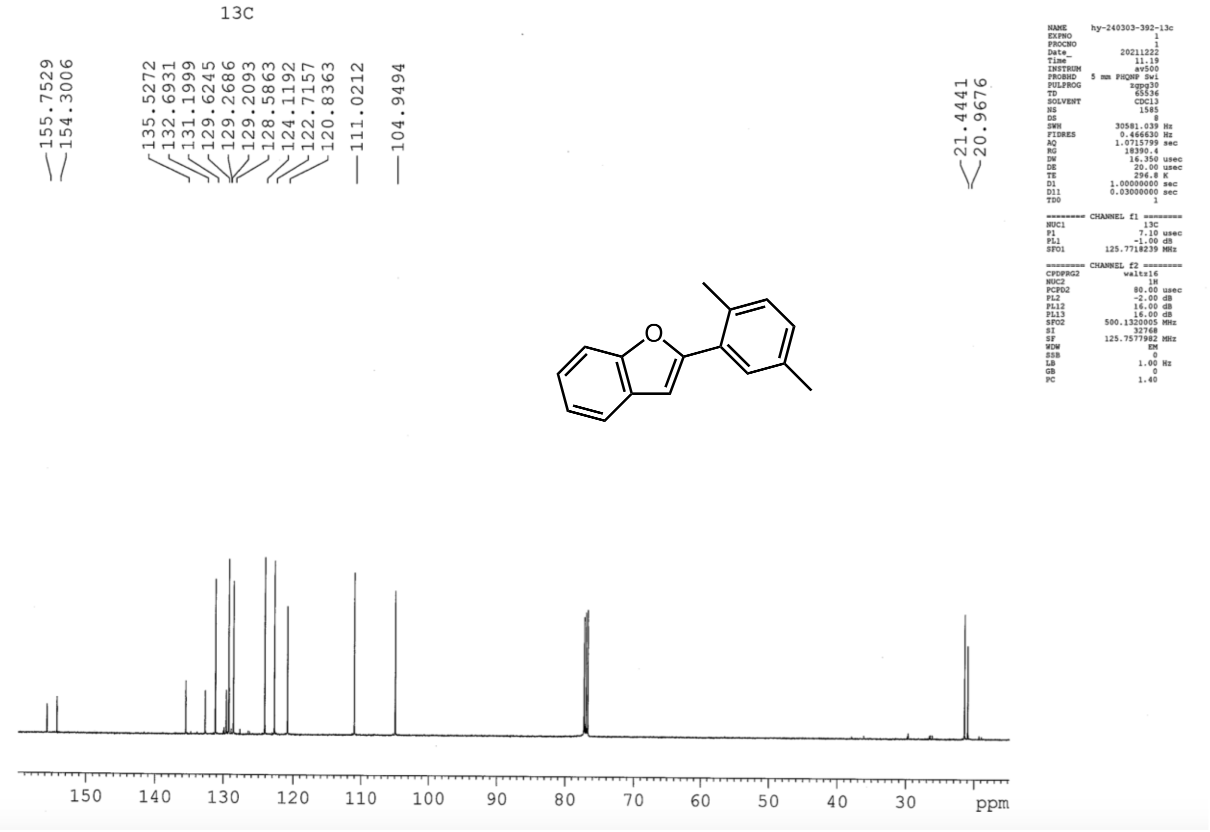

2-[4-(*tert*-Butyl)phenyl]benzofuran (3ad)

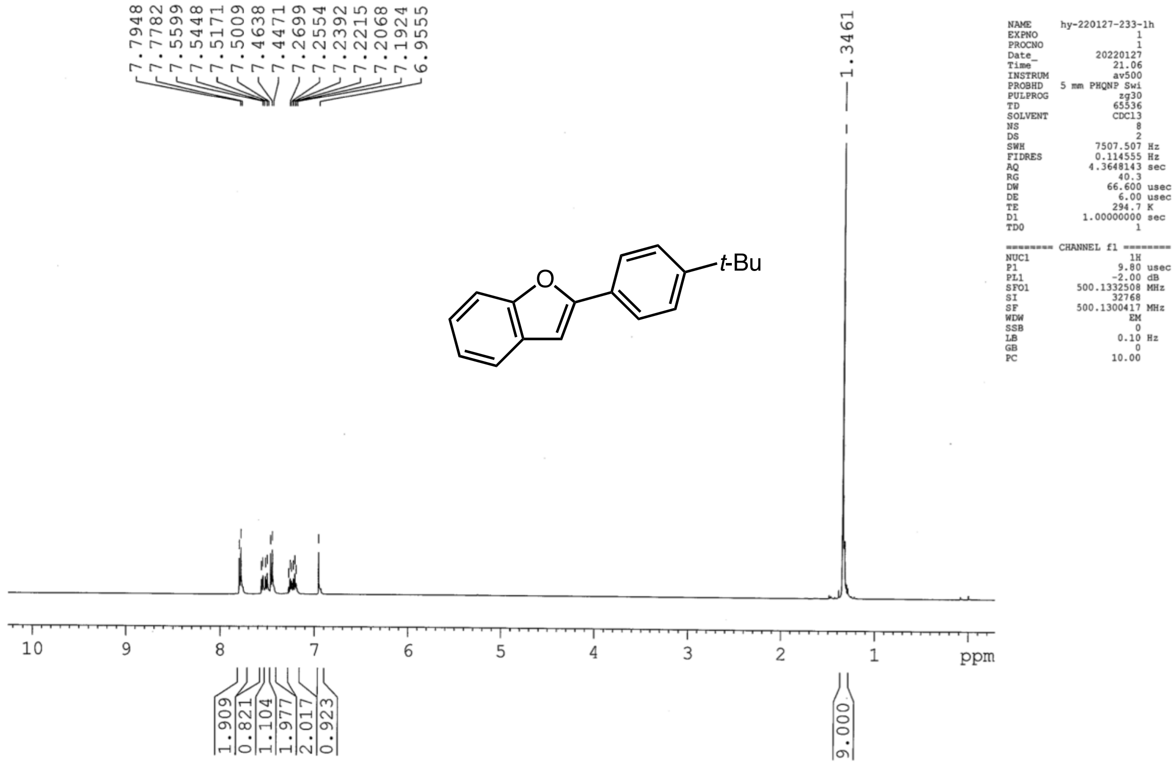

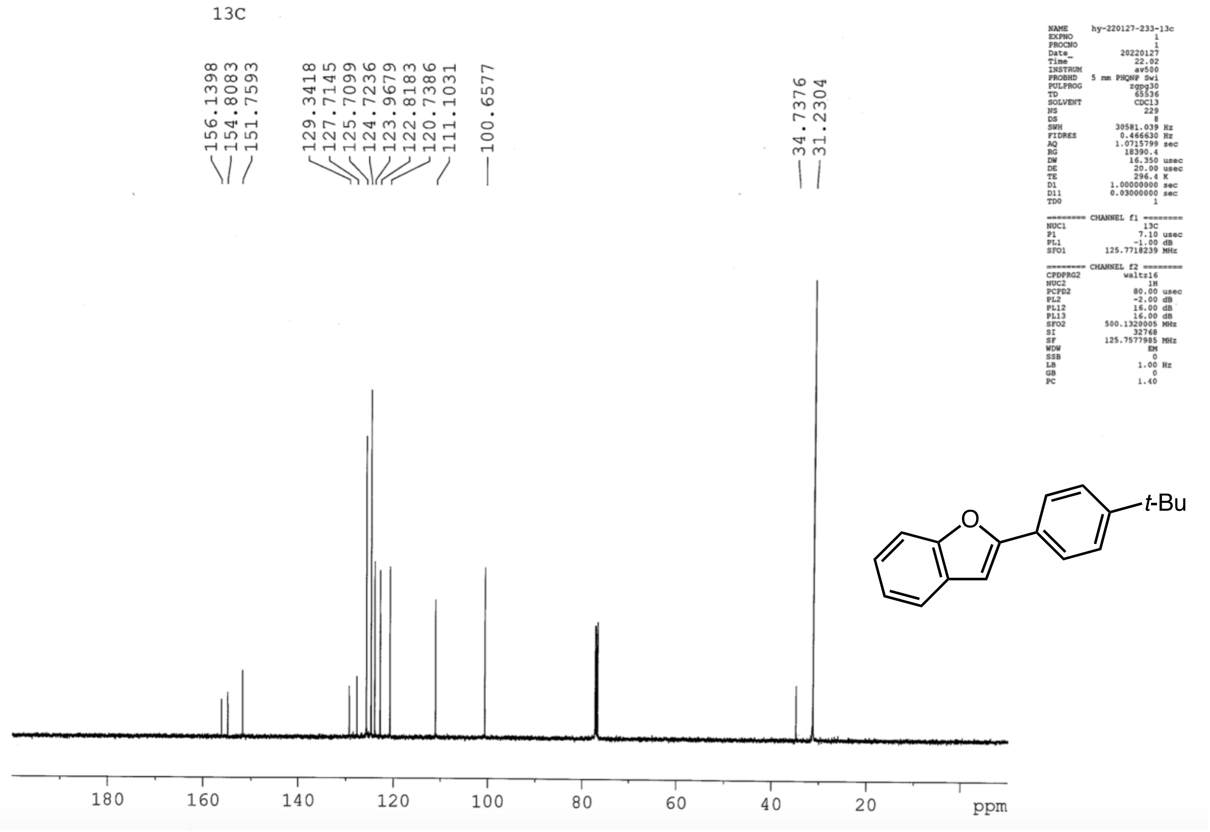

2-(3,5-Dimethoxyphenyl)benzofuran (3ae)

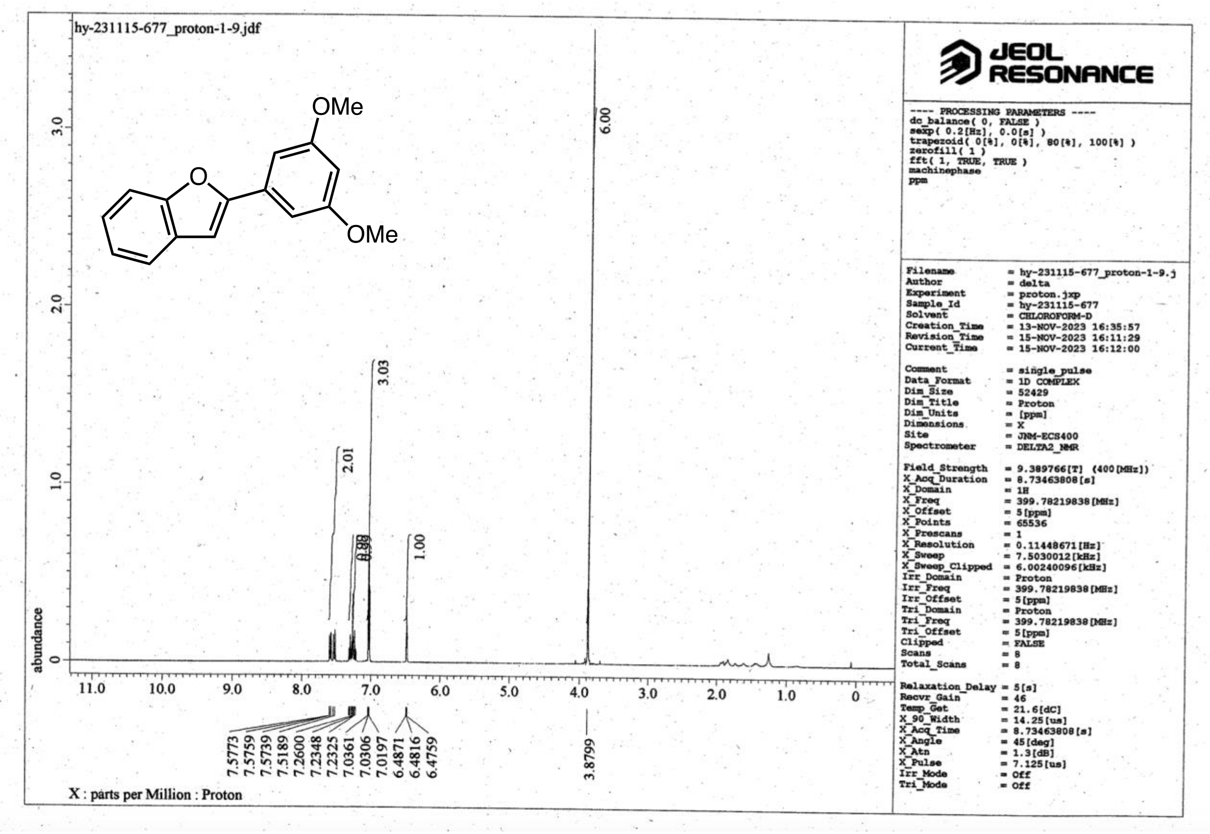

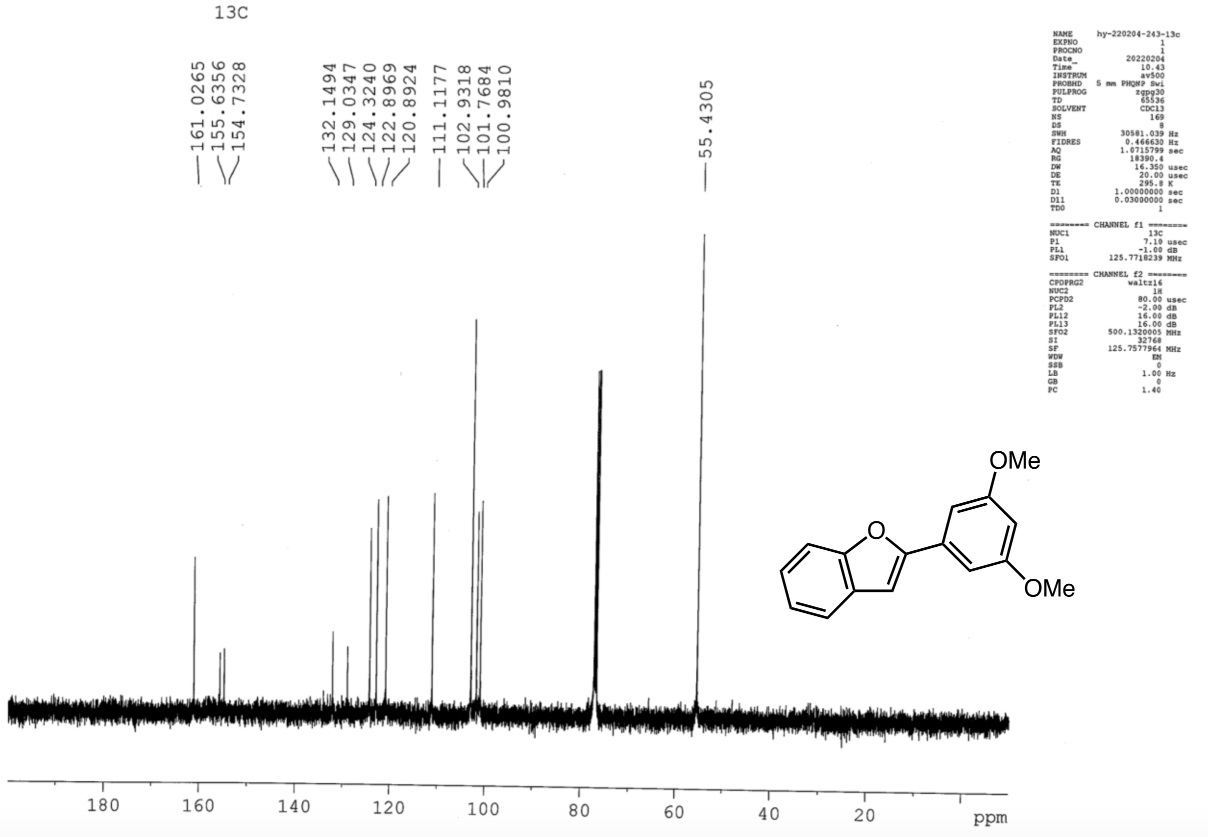

2-Phenylnaphtho[2,1-*b*]furan (3ba)

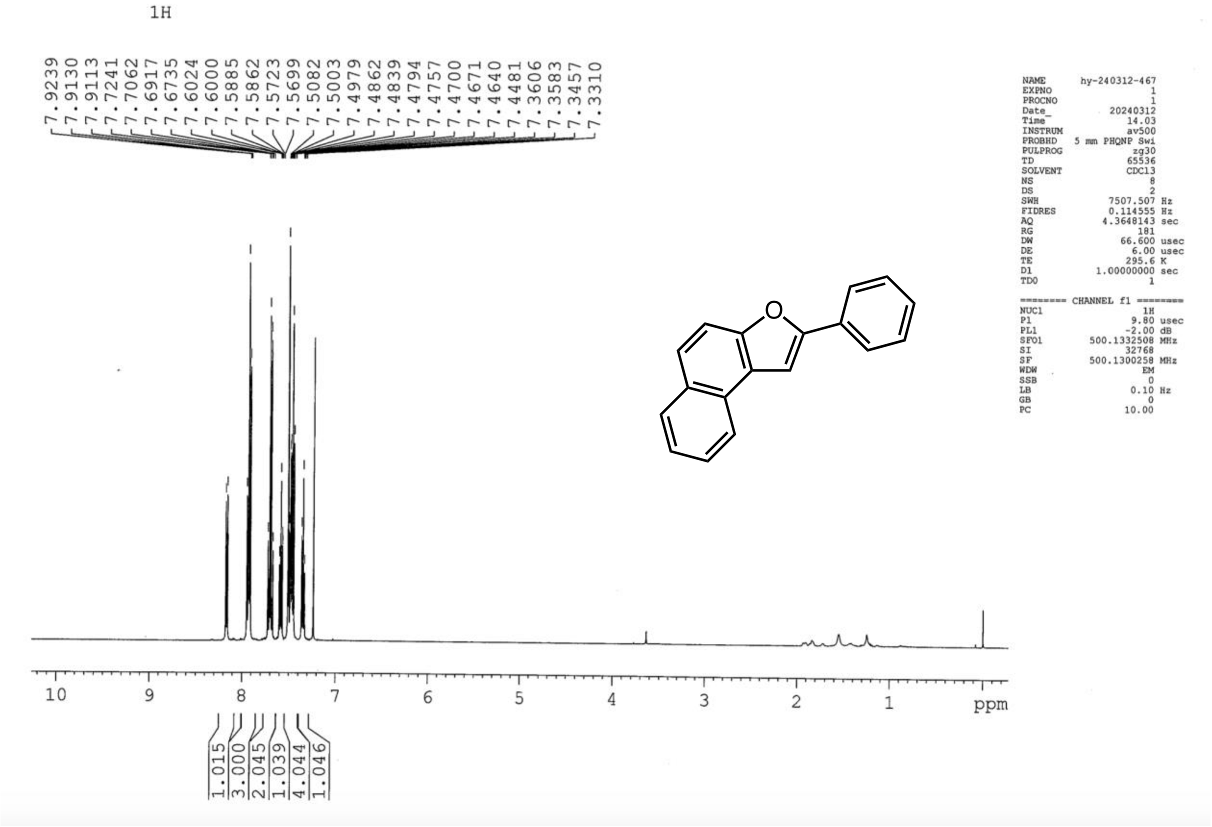

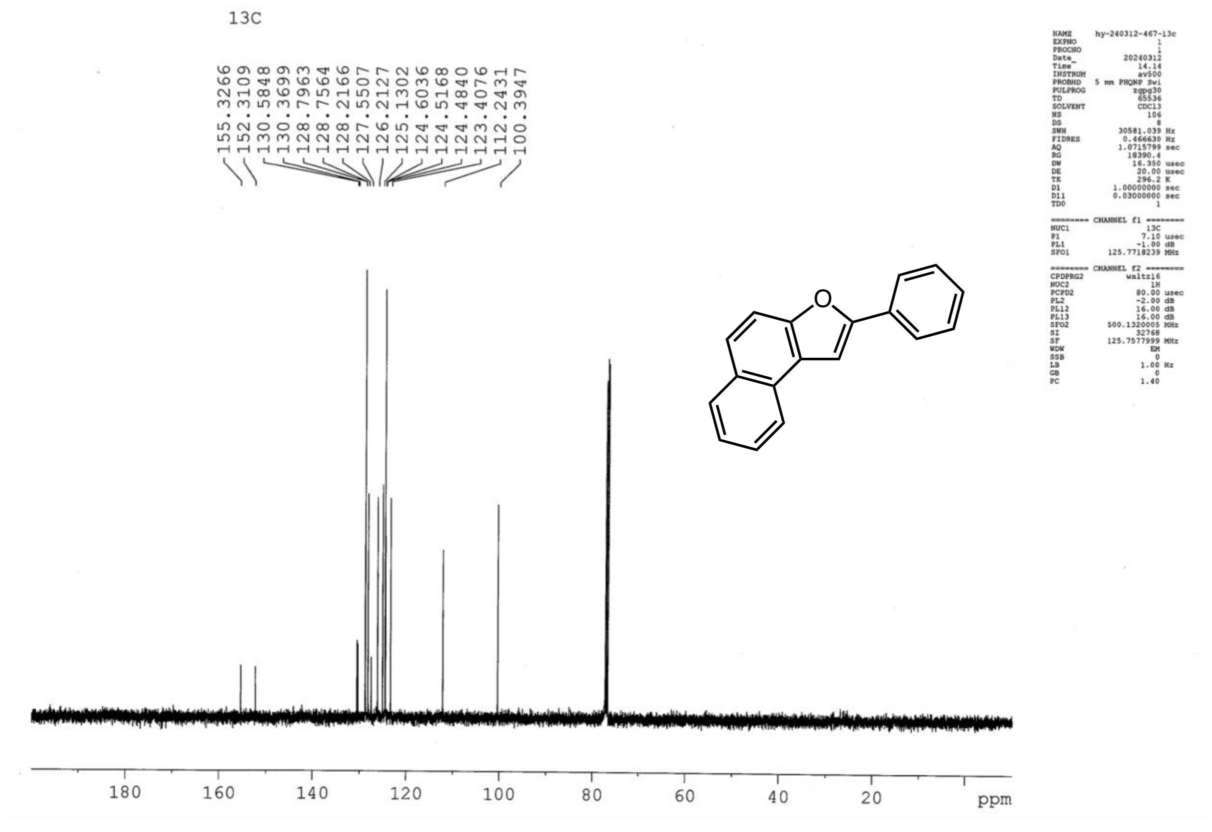

2-(3-Methylphenyl)naphtho[2,1-*b*]furan (3bb)

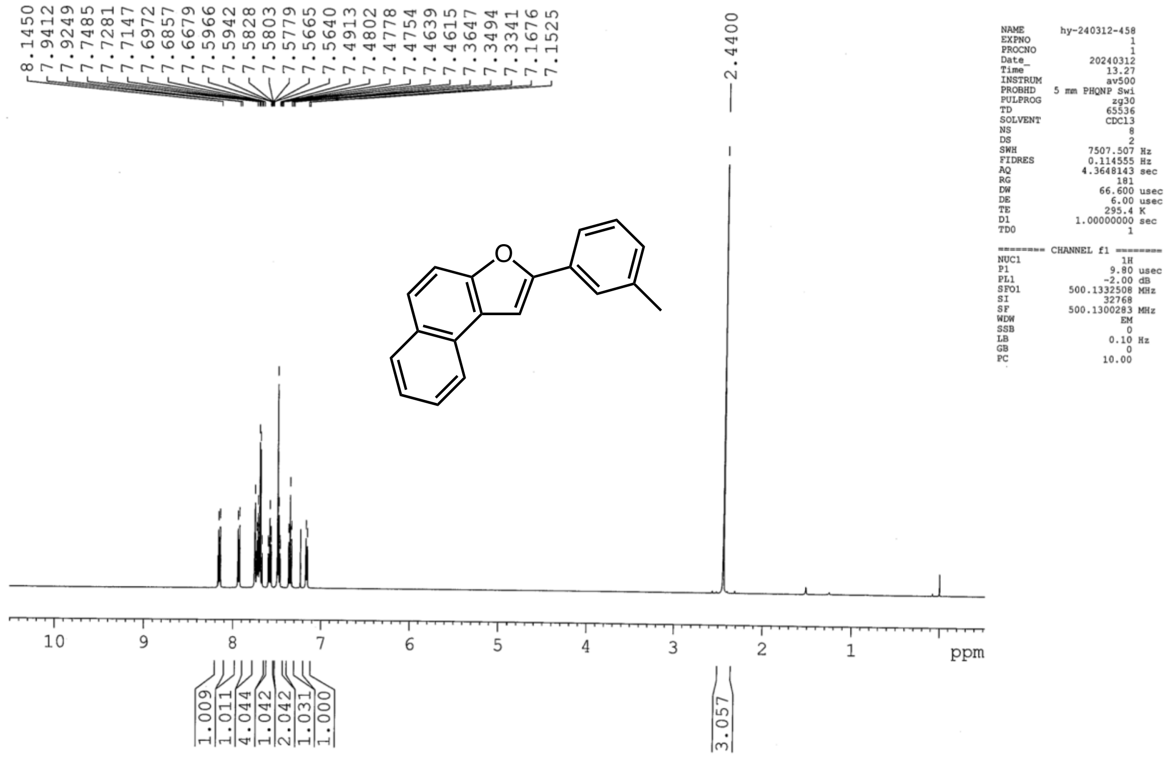

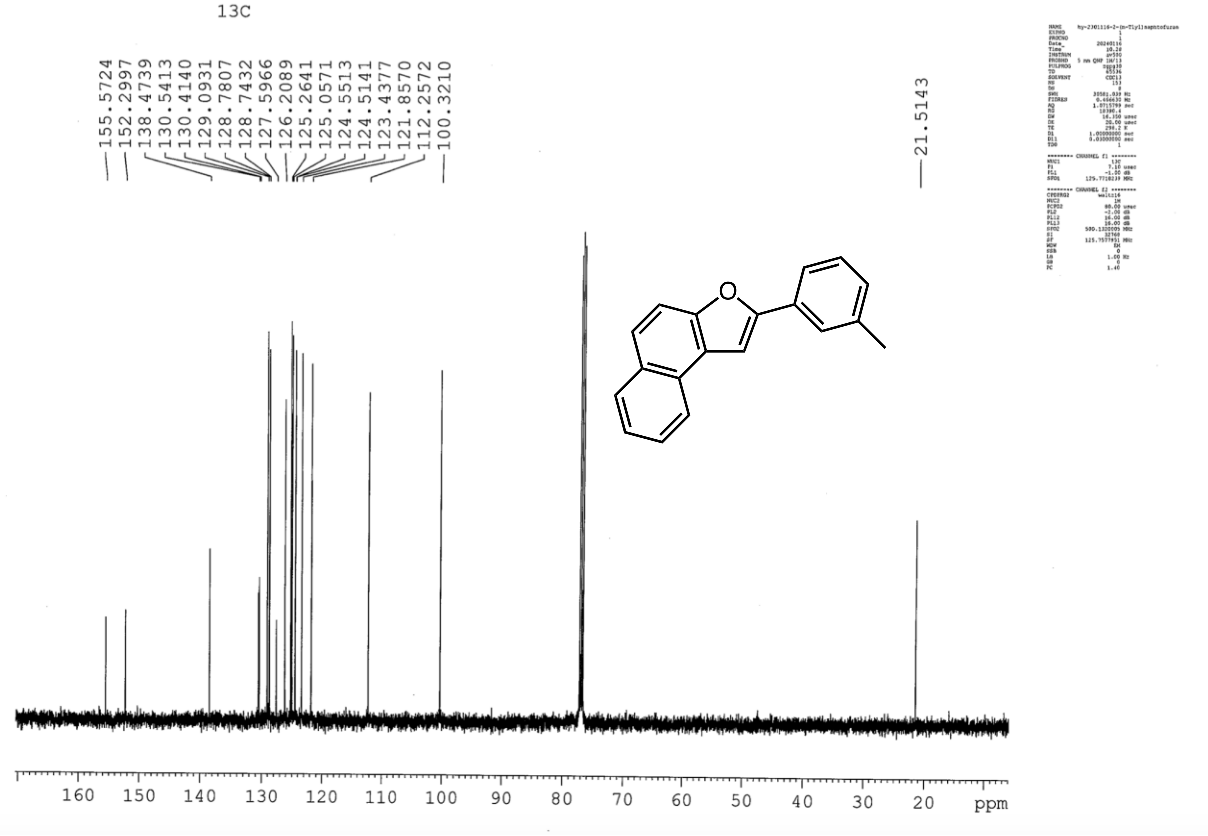

2-[4-(*tert*-Butyl)phenyl]naphtho[2,1-*b*]furan (3bd)

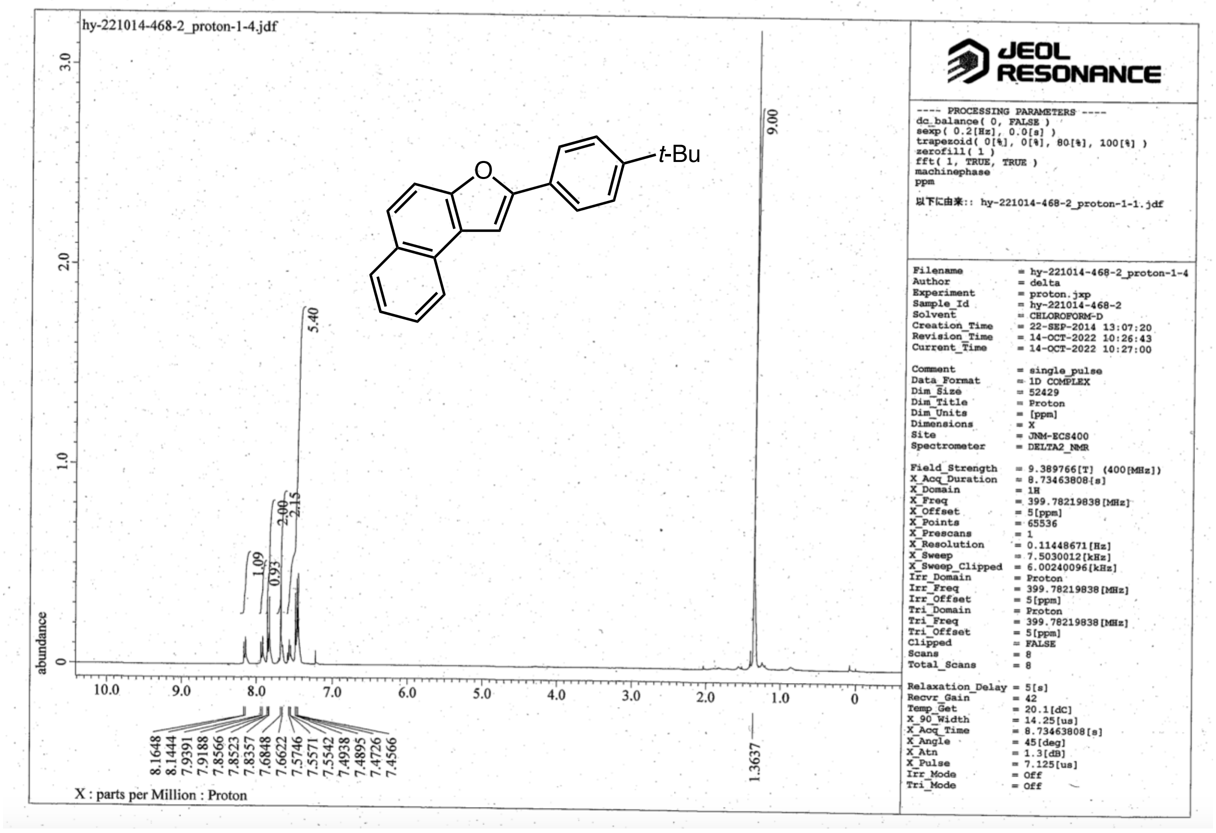

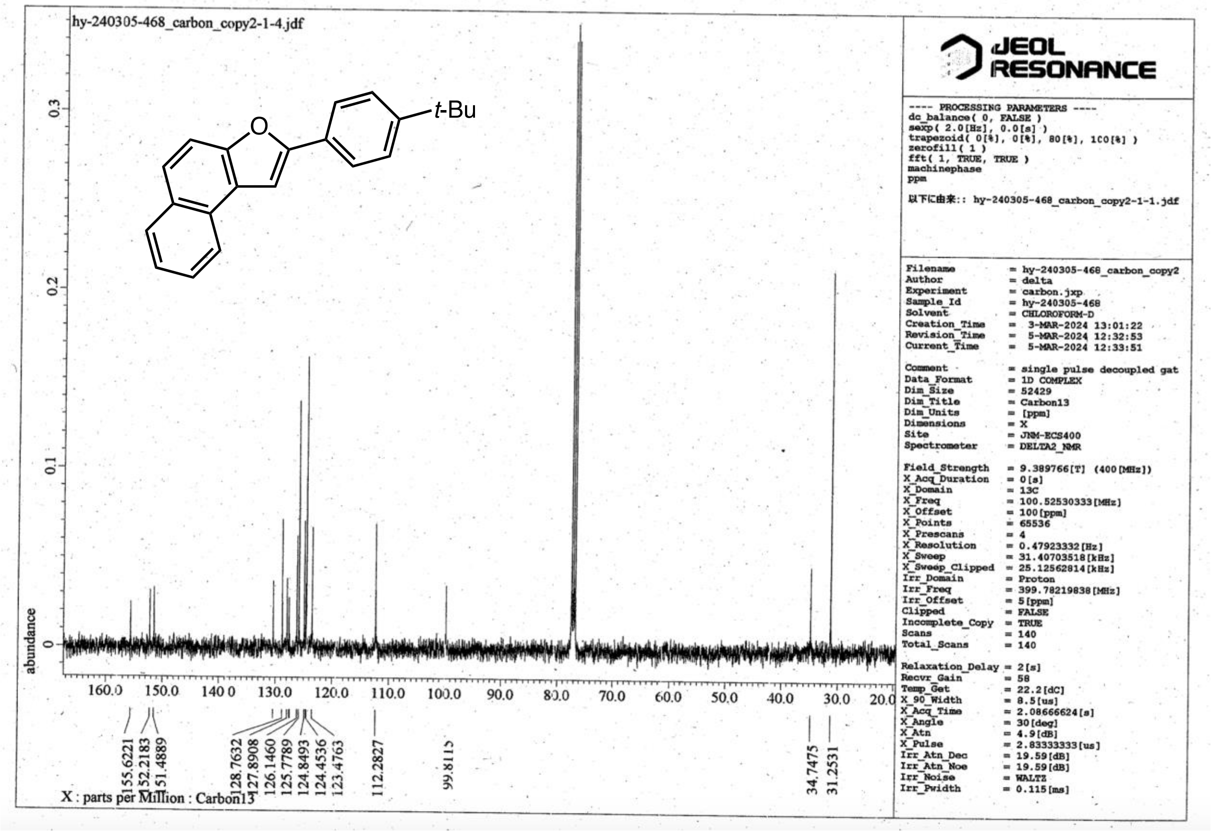

2-(4-Methoxyphenyl)naphtho[2,1-*b*]furan (3bf)

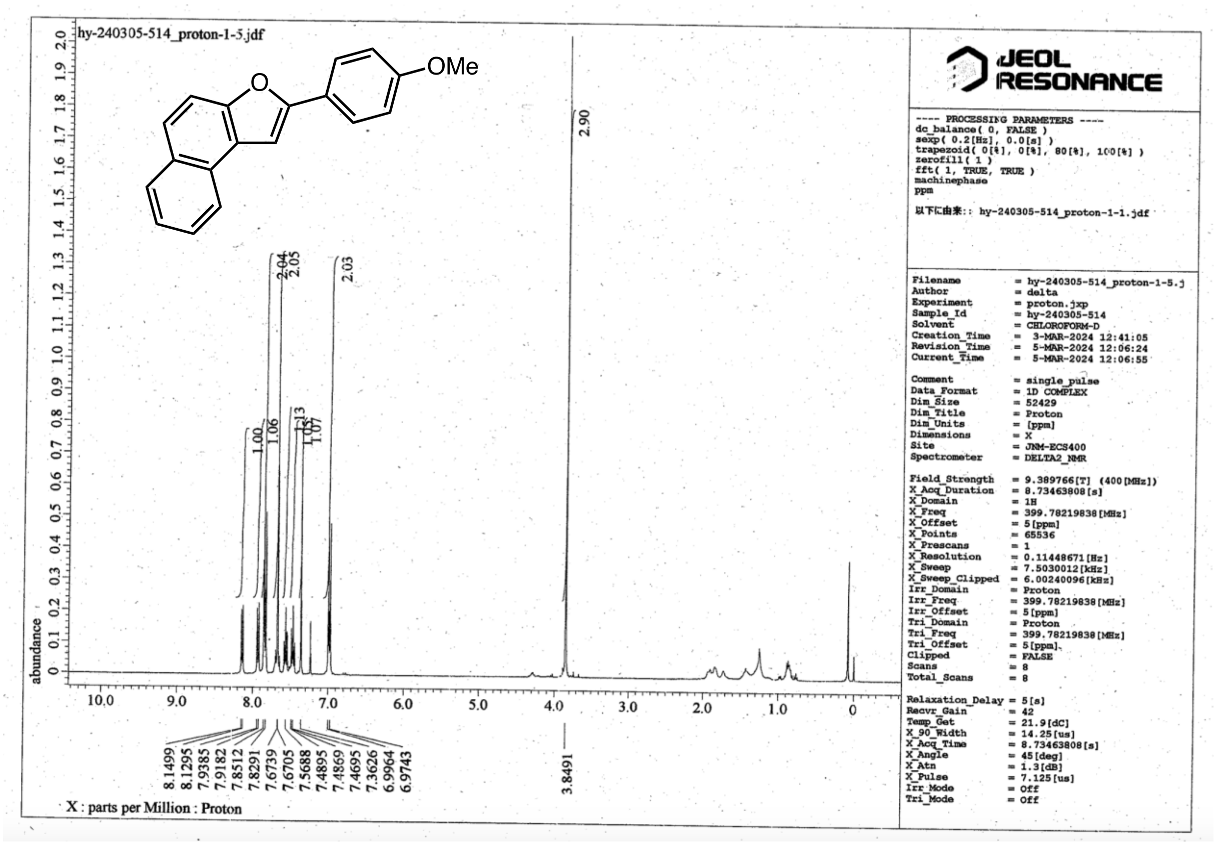

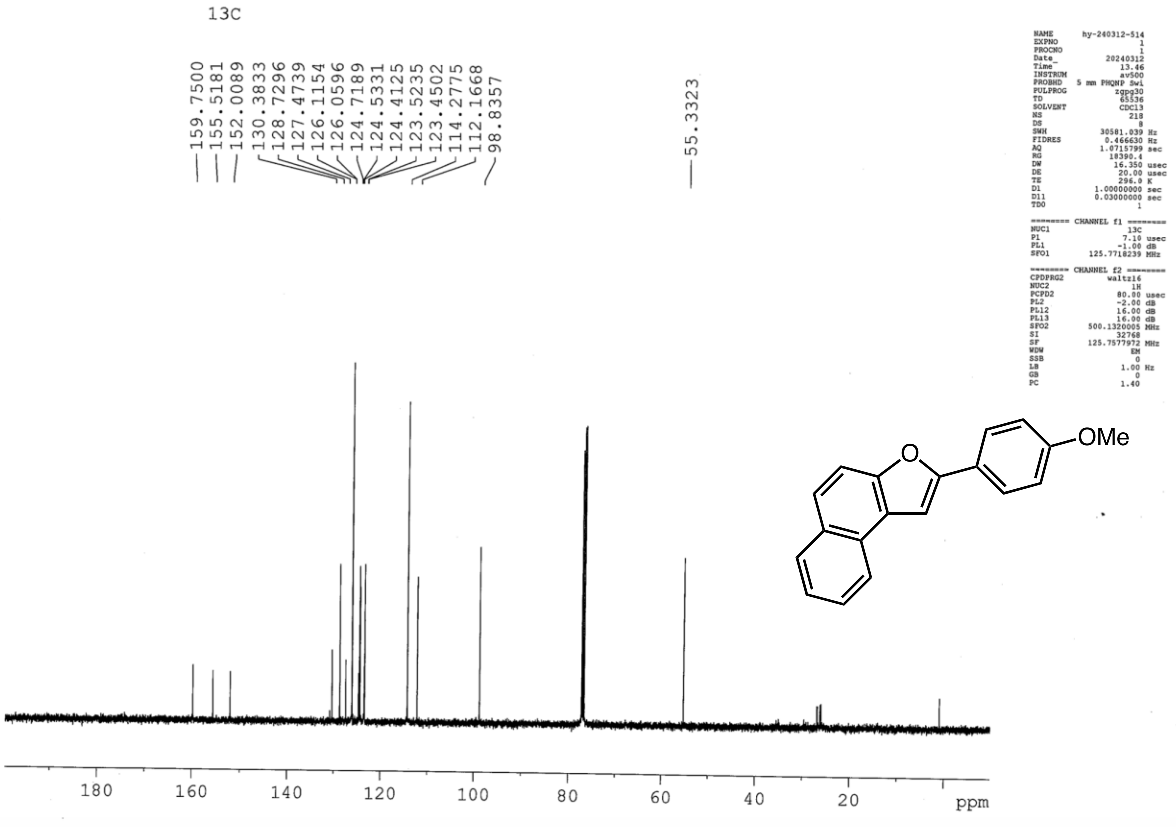

2-[4-(Trifluoromethyl)phenyl]naphtho[2,1-*b*]furan (3bg)

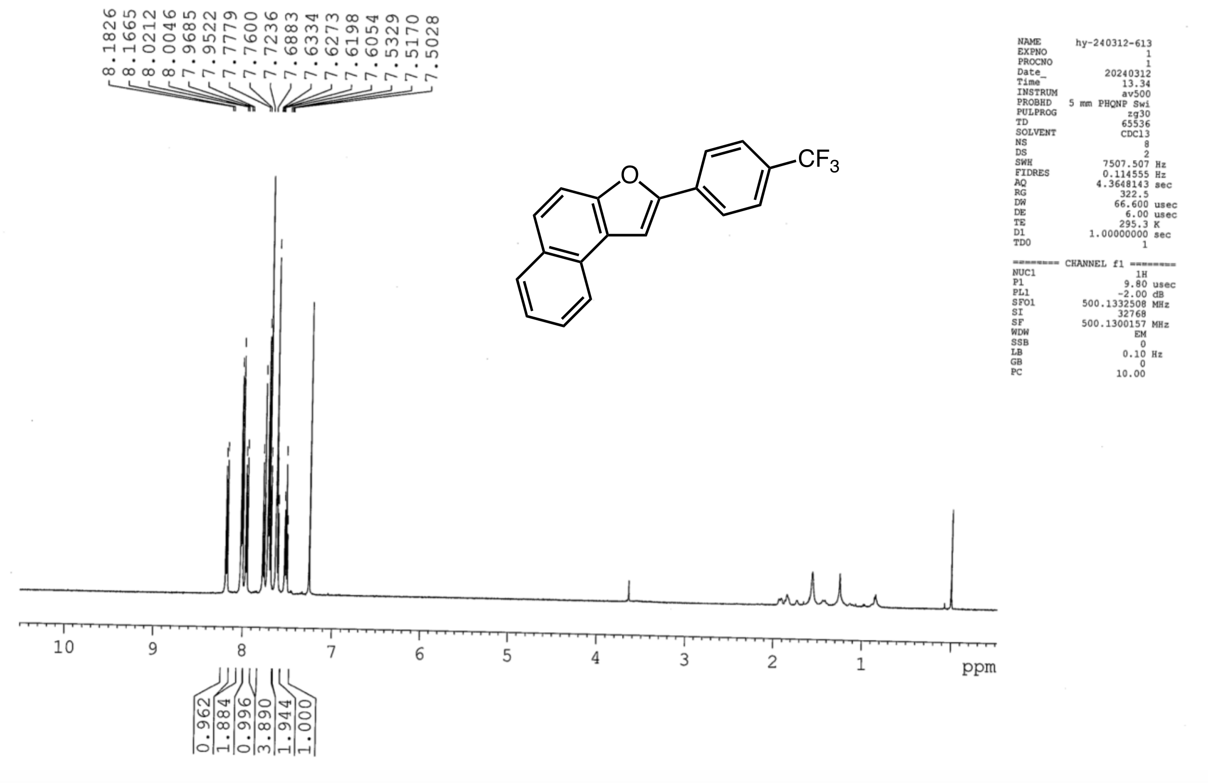

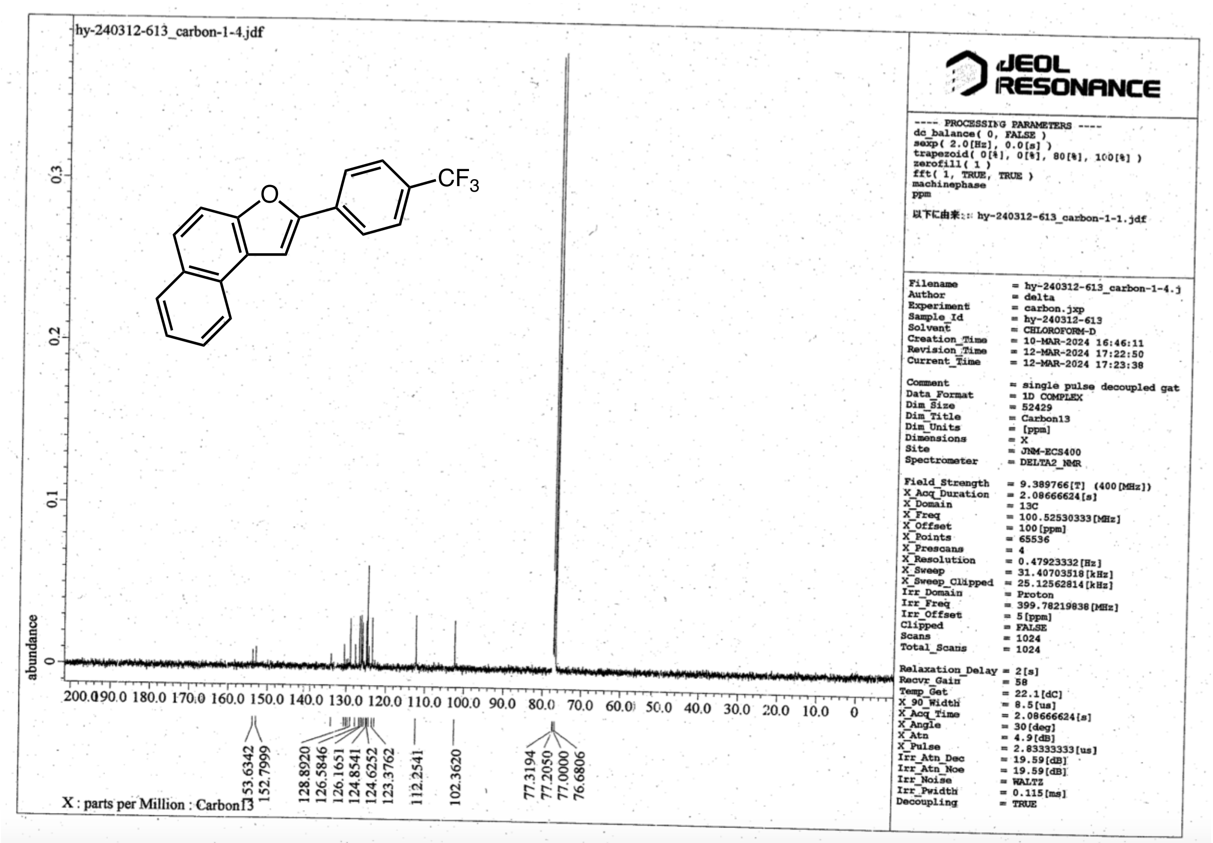

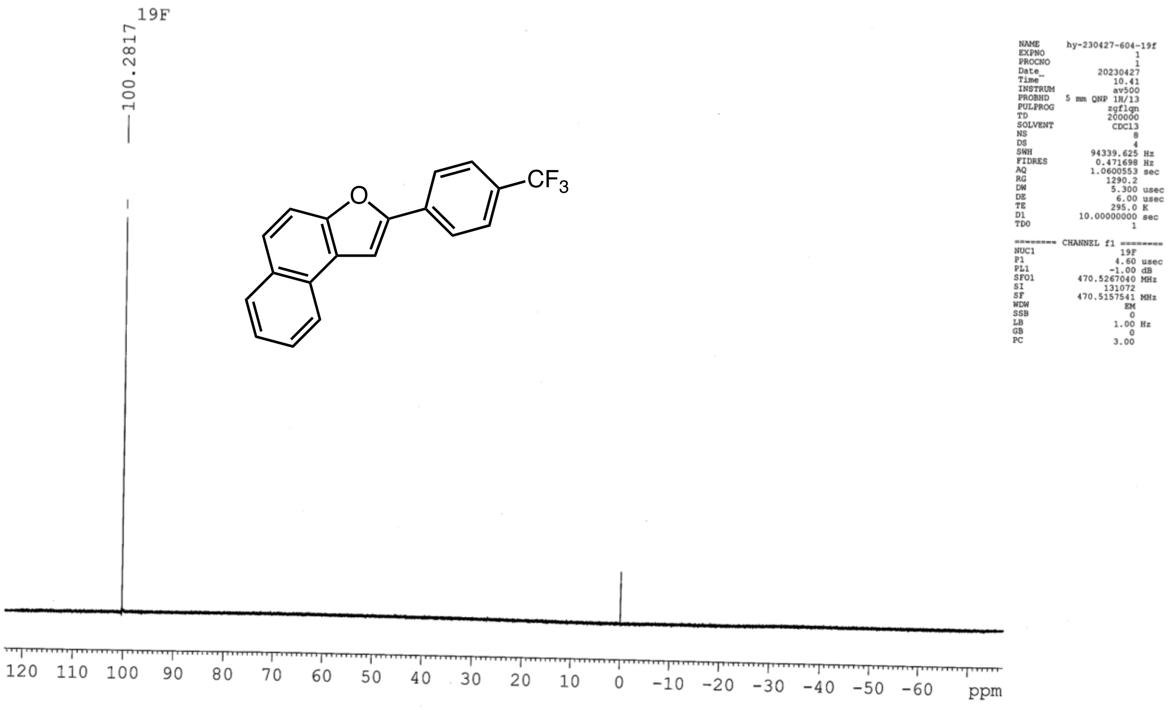
19F
100.2817
O
CF3
NAME hy-230427-604-19f
EXPNO 1
PROCNO 1
Date_ 20230427
Time 10.41
INSTRUM av500
PROBHD 5 mm QNP 1H/13
PULPROG zgflqn
TD 200000
SOLVENT CDCl3
NS 8
DS 4
SWH 94339.625 Hz
FIDRES 0.471698 Hz
AQ 1.0600553 sec
RG 1290.2
DW 5.300 usec
DE 6.00 usec
TE 295.0 K
D1 10.00000000 sec
TD0 1
======== CHANNEL f1 ========
NUC1 19F
P1 4.60 usec
PL1 -1.00 dB
SFO1 470.5267040 MHz
SI 131072
SF 470.5157541 MHz
WDW EM
SSB 0
LB 1.00 Hz
GB 0
PC 3.00
120 110 100 90 80 70 60 50 40 30 20 10 0 -10 -20 -30 -40 -50 -60 ppm

Ethyl 4-(naphtho[2,1-*b*]furan-2-yl)benzoate (3bh)

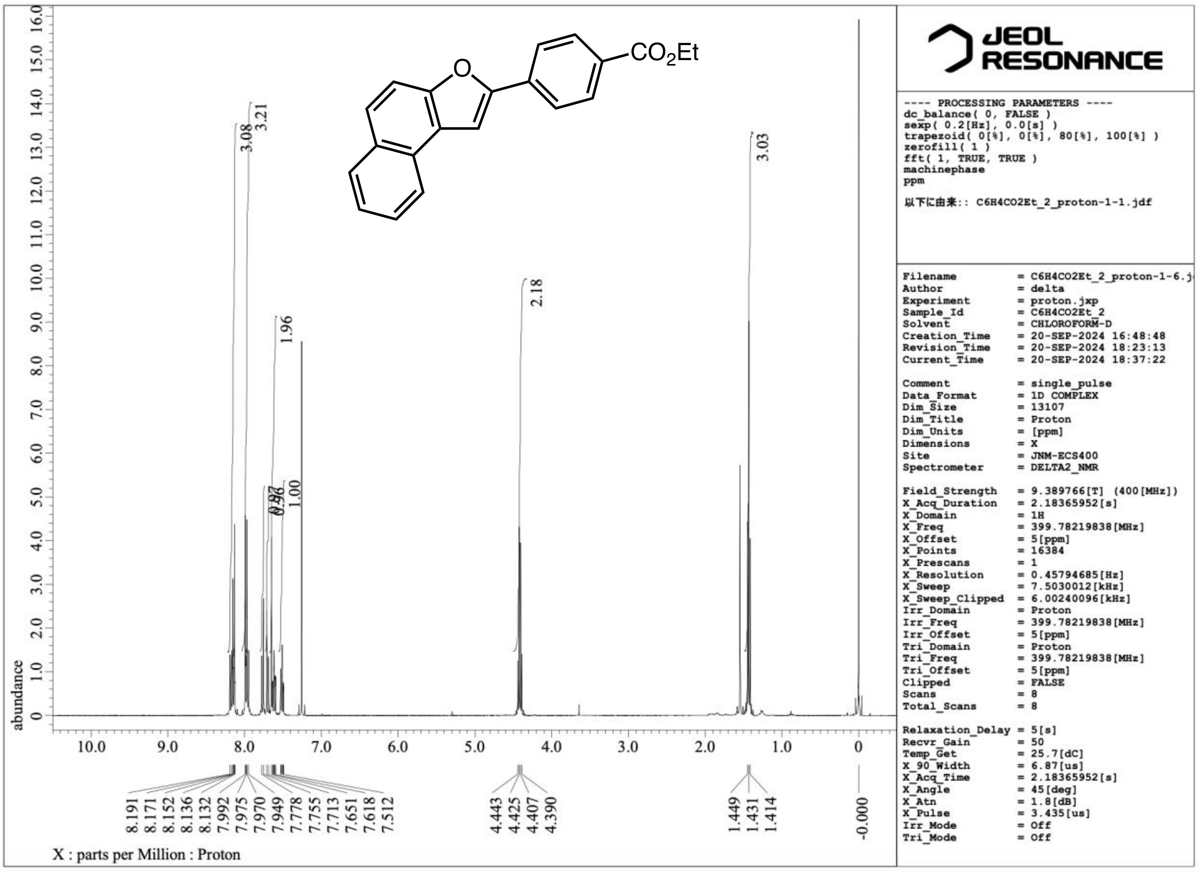

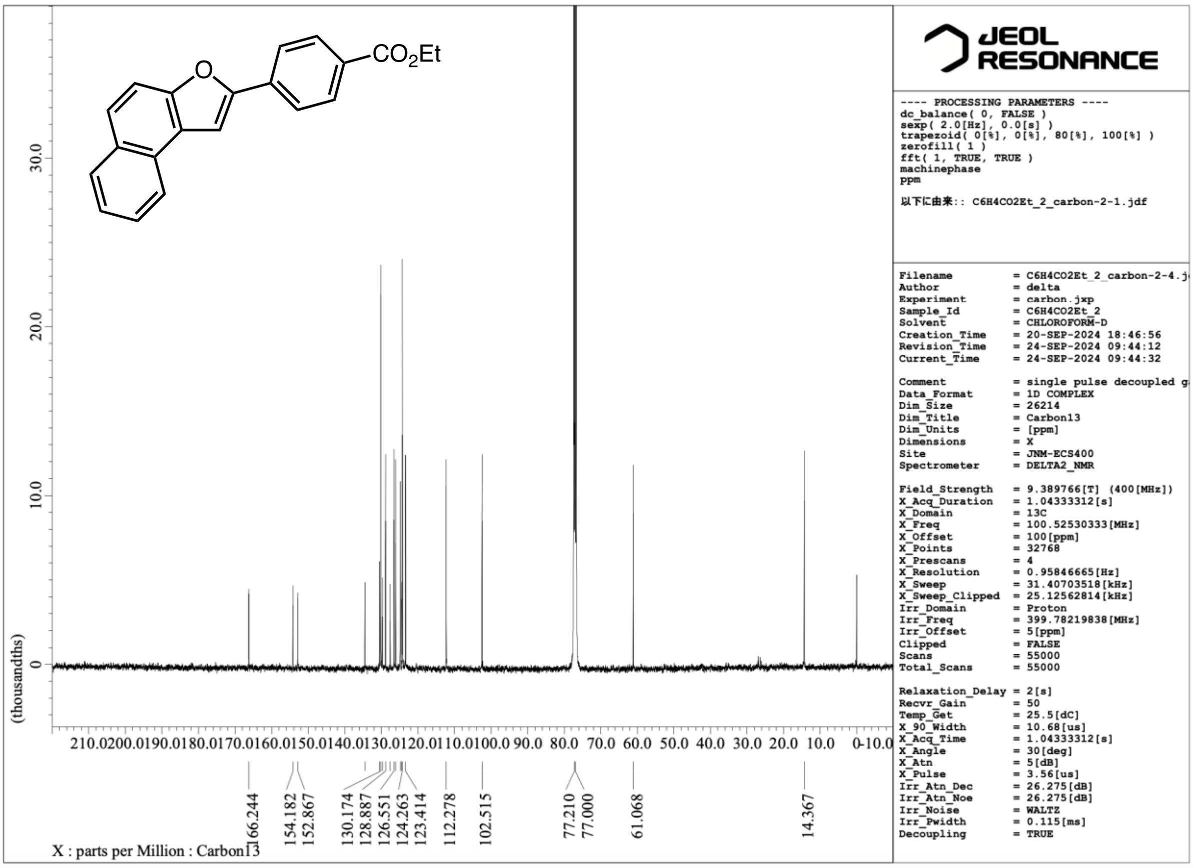

2-(Naphthalen-2-yl)naphtho[2,1-*b*]furan (3bi)

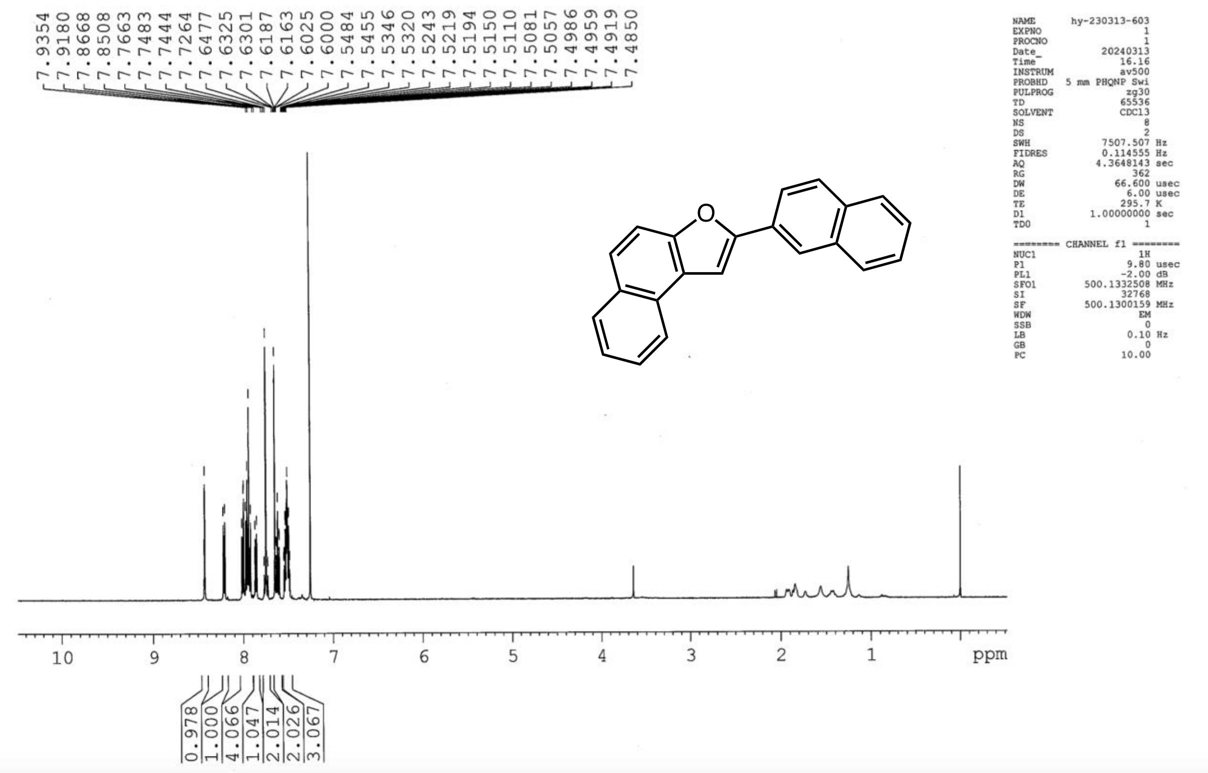

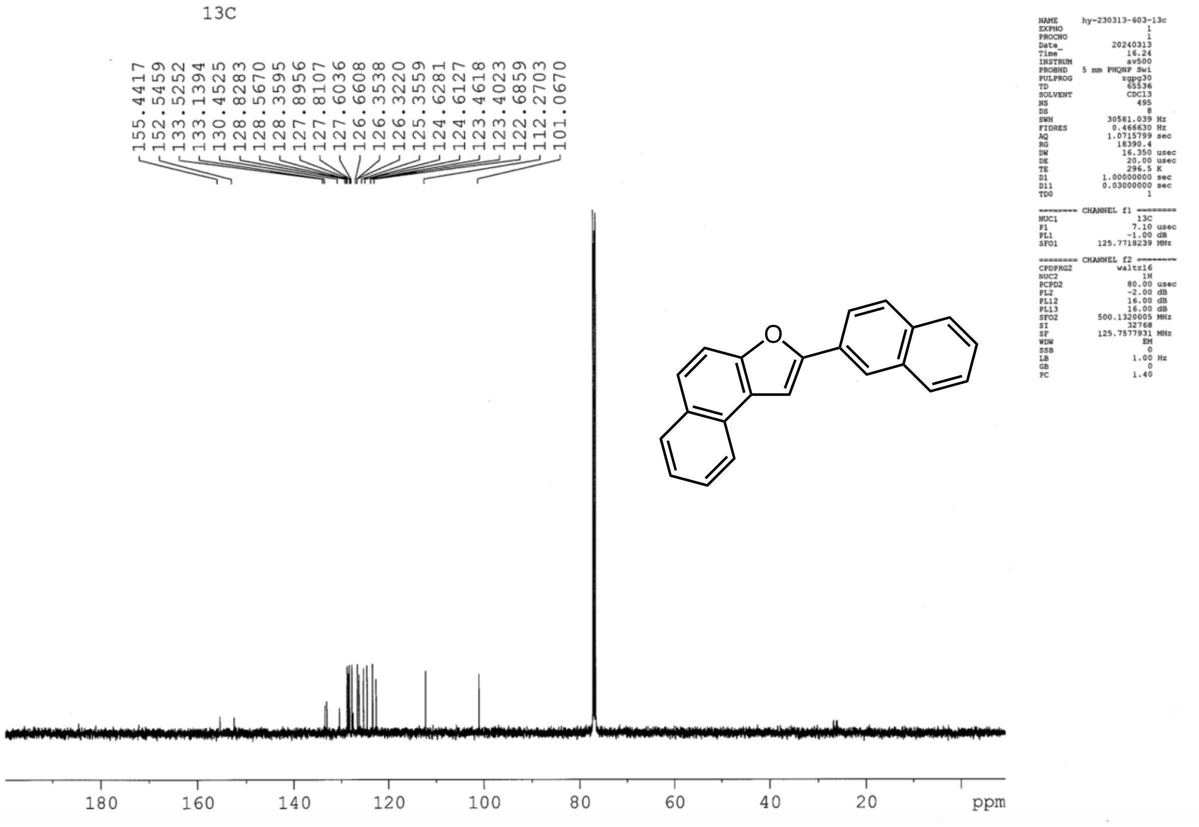

7-Methoxy-2-phenylbenzofuran (3ca)

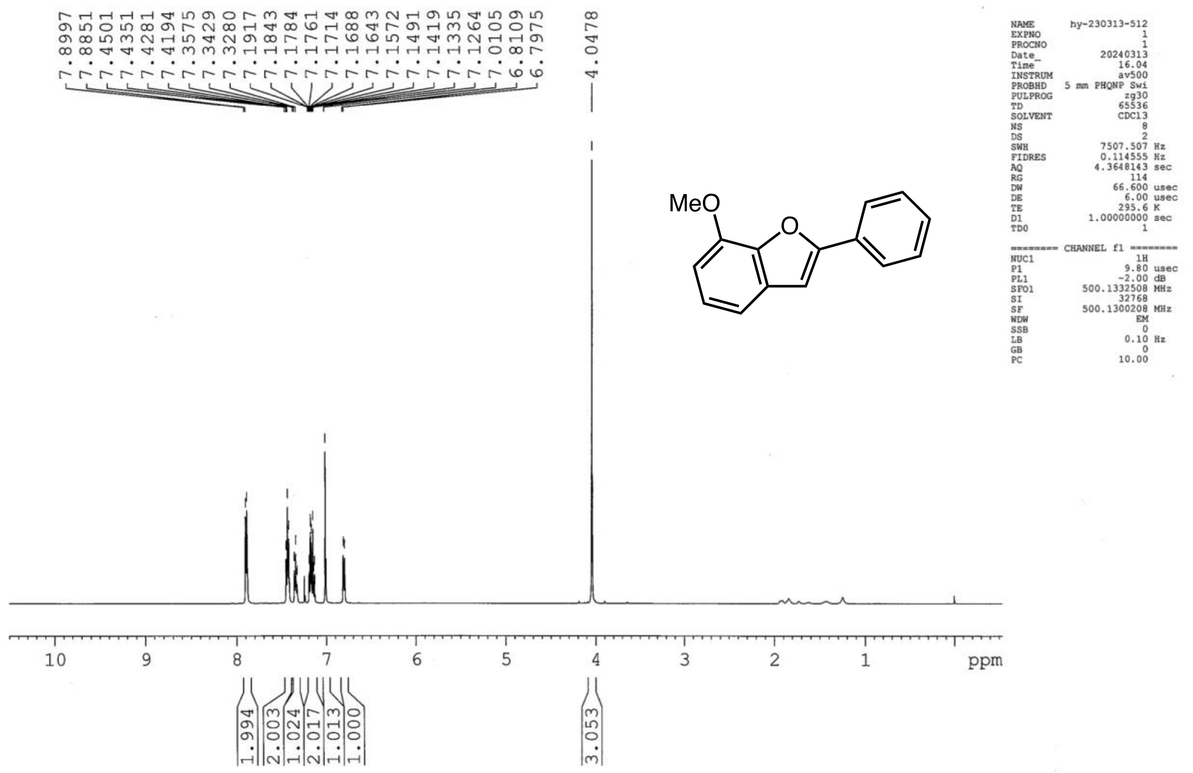

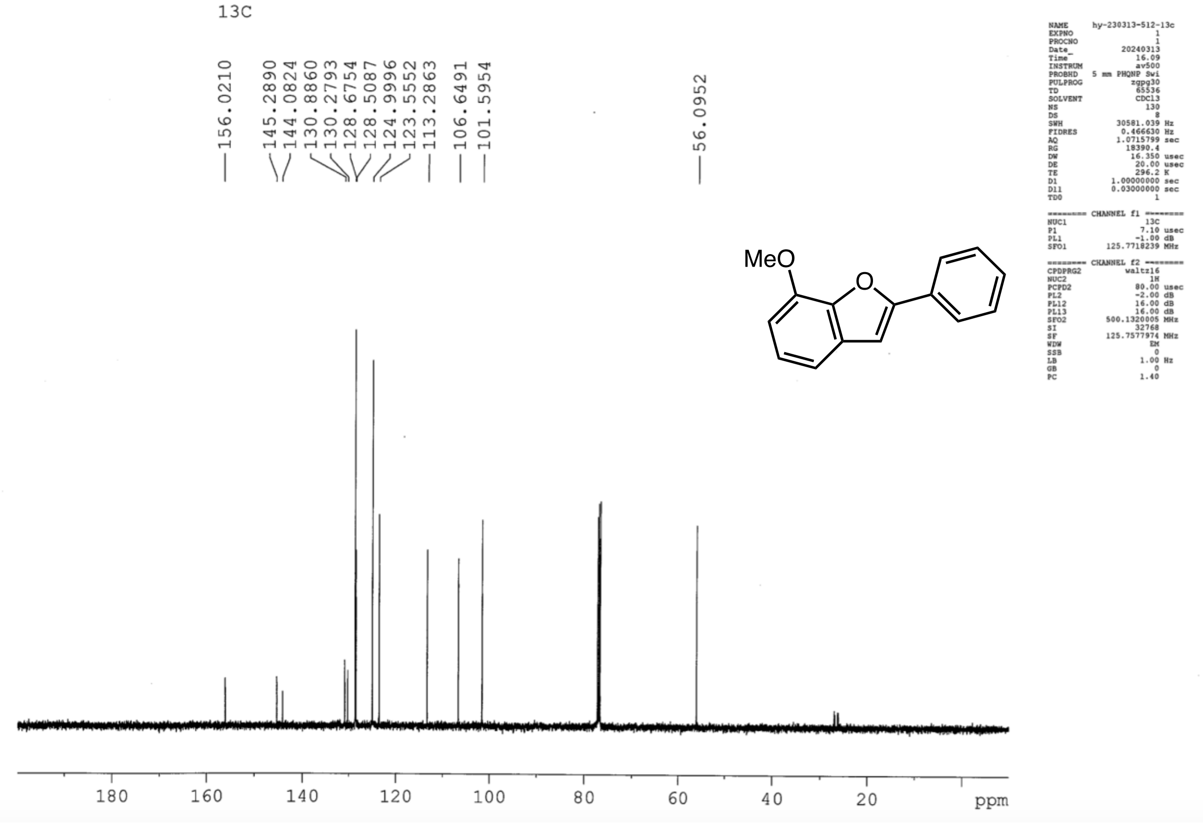

7-Ethoxy-2-phenylbenzofuran (3da)

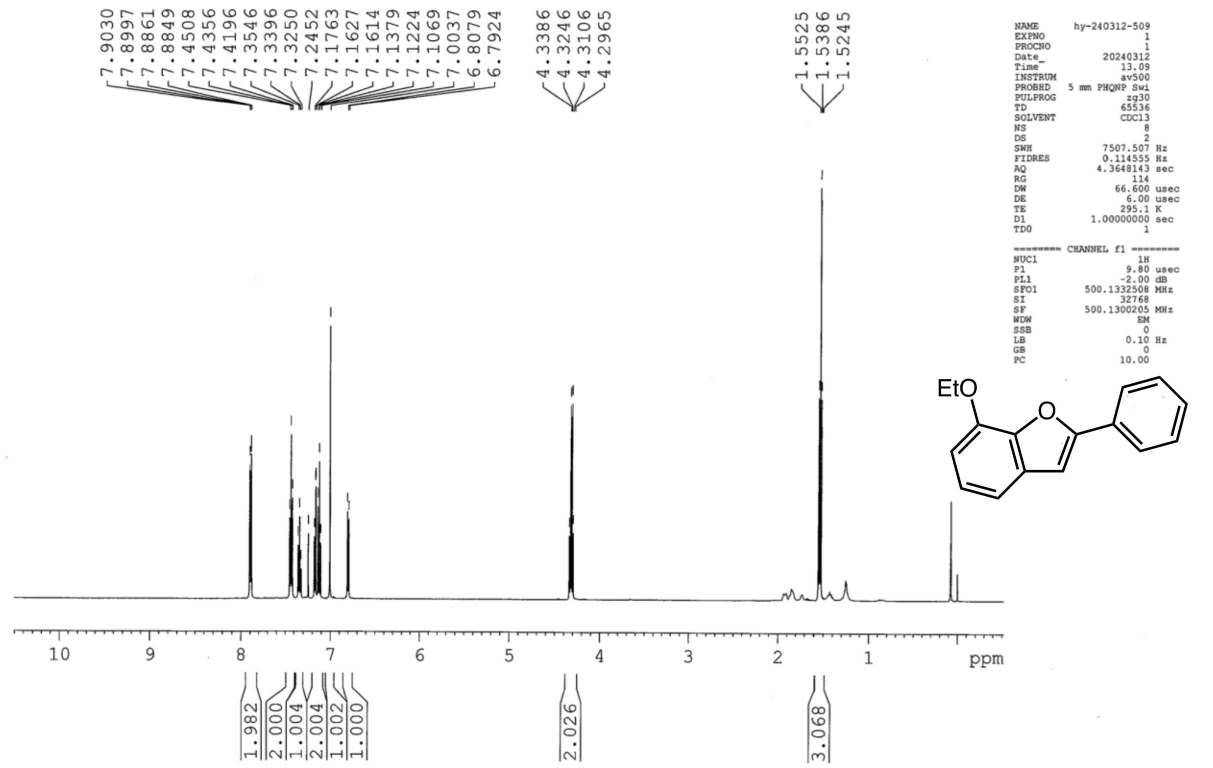

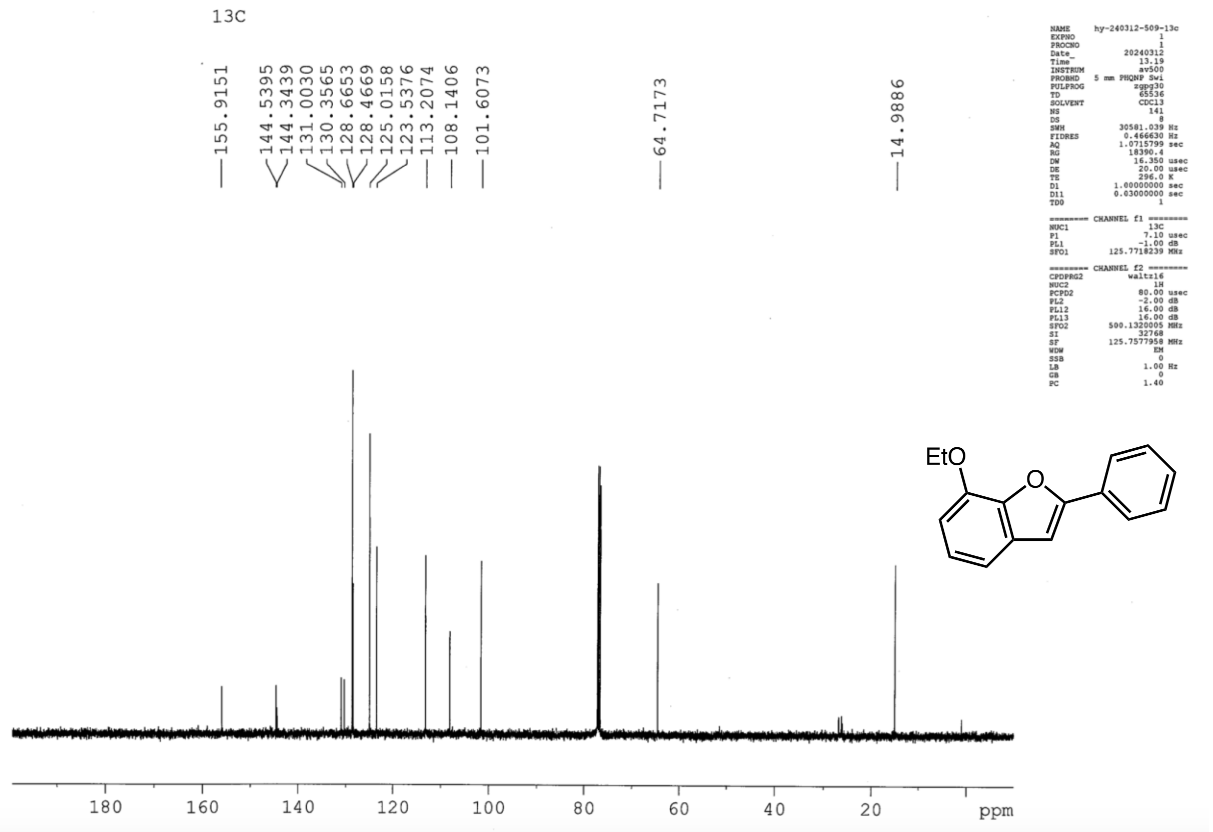

2-Phenylbenzo[*b*]thiophene (5)

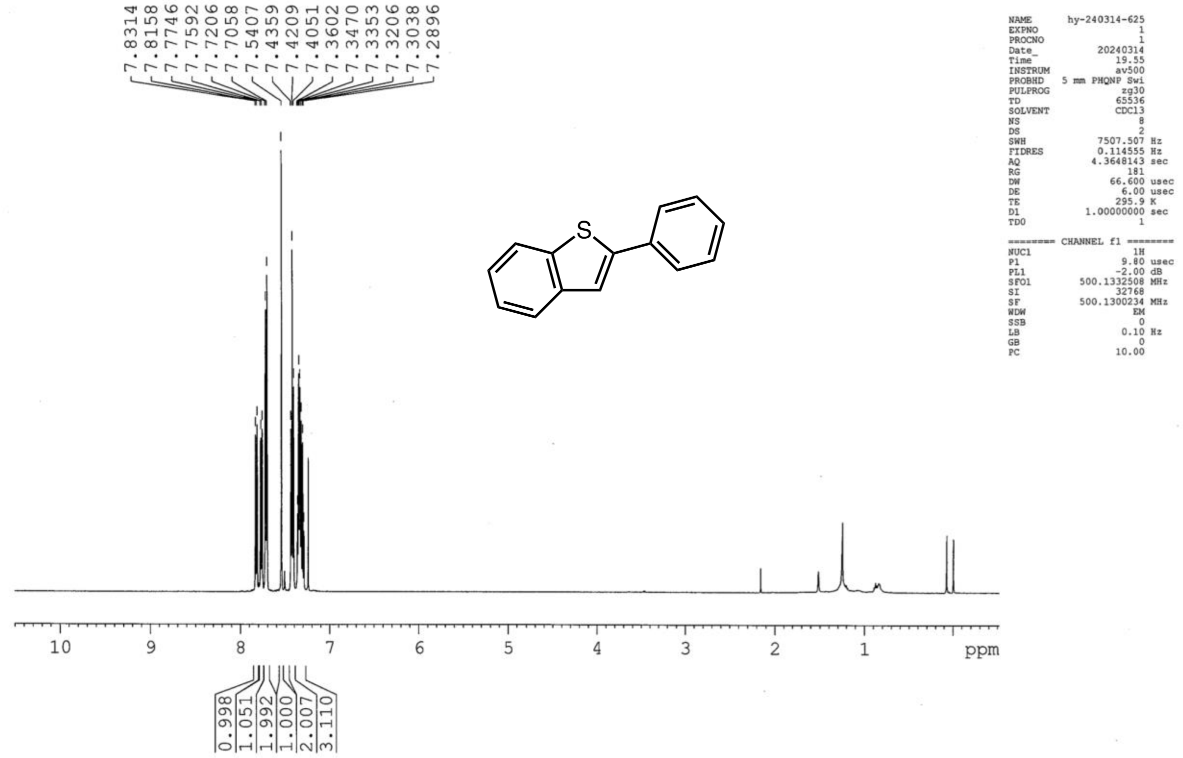

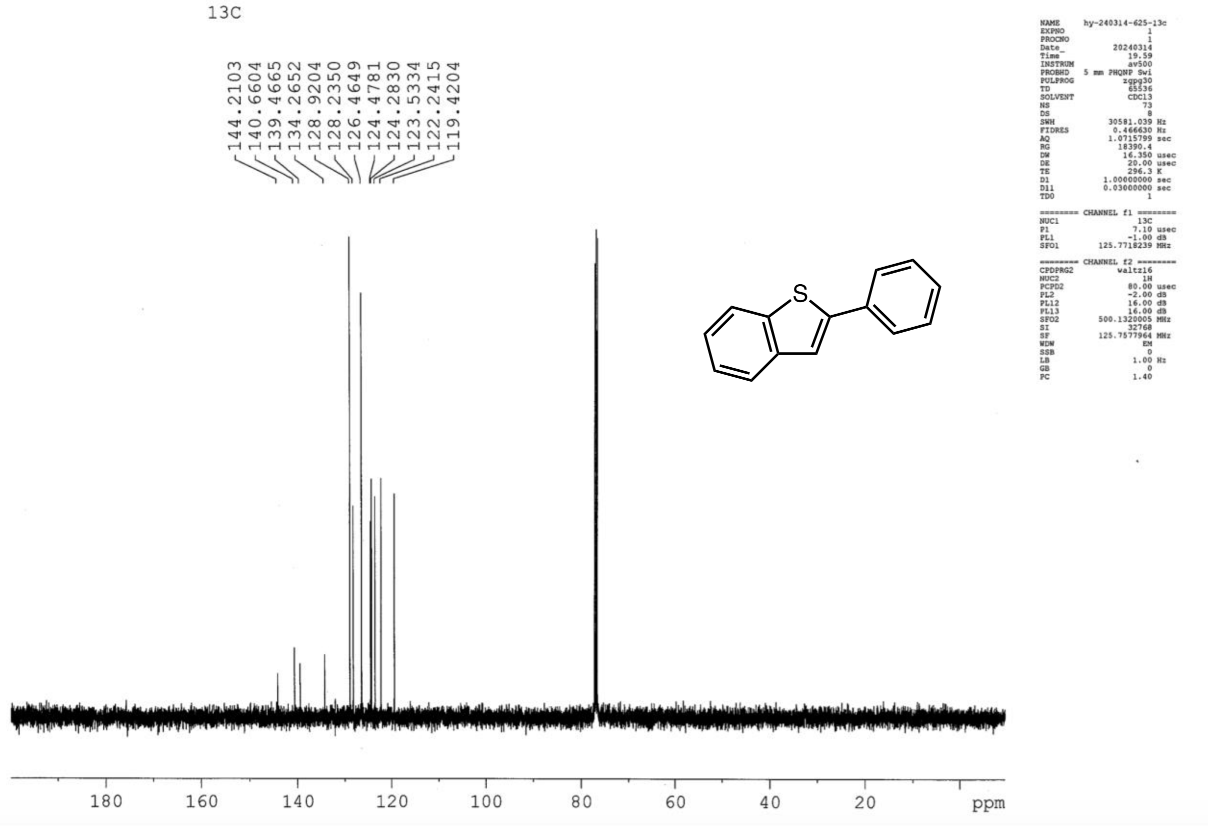

2-Fluoro-5-[4-(trifluoromethyl)phenyl]benzofuran (1f)

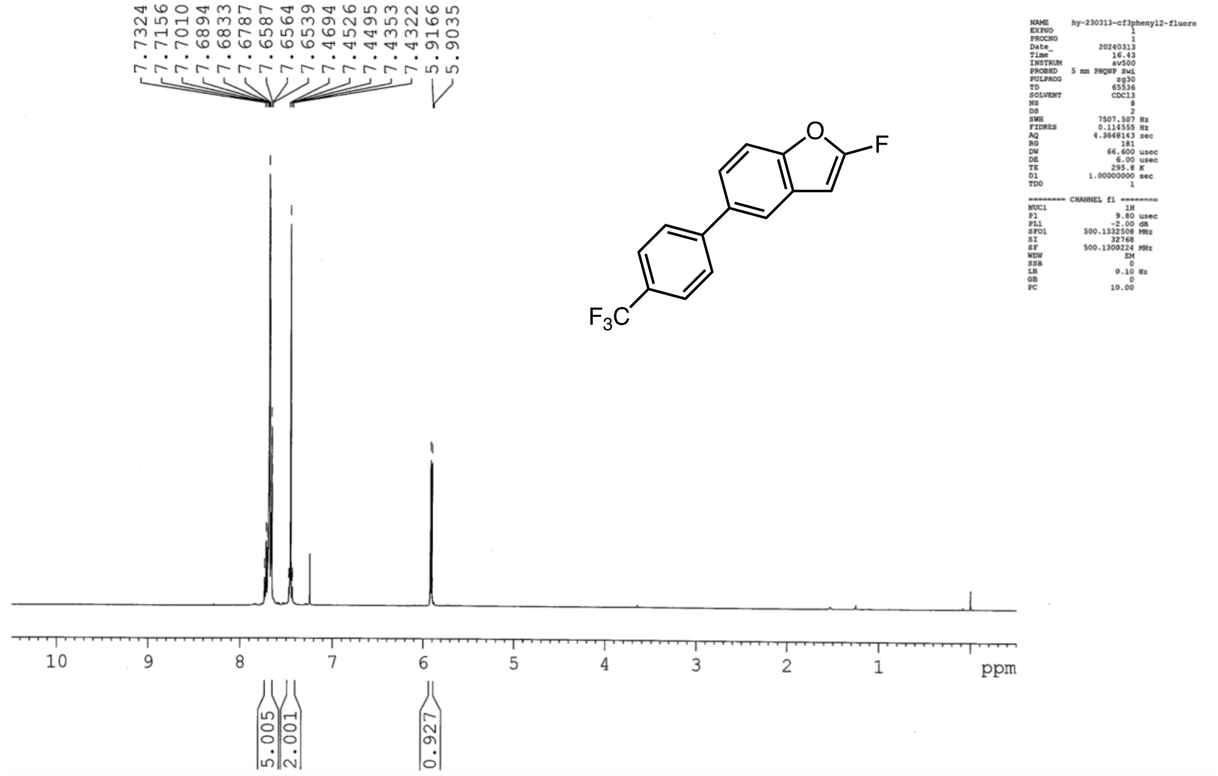

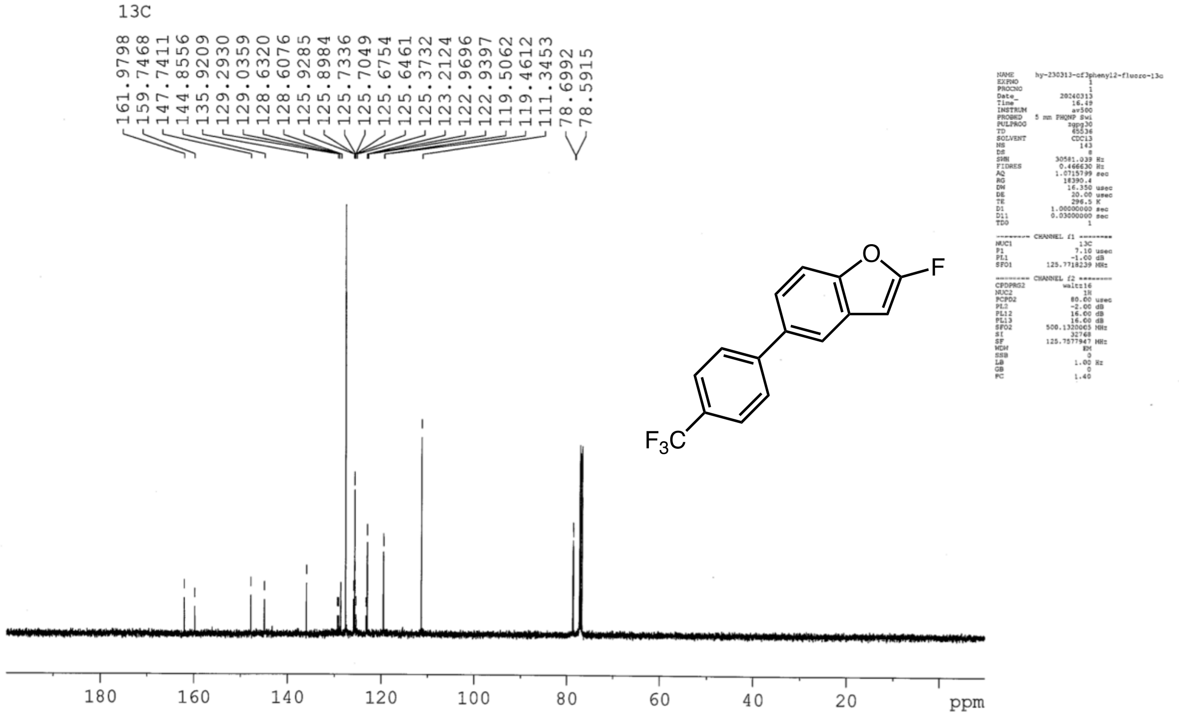

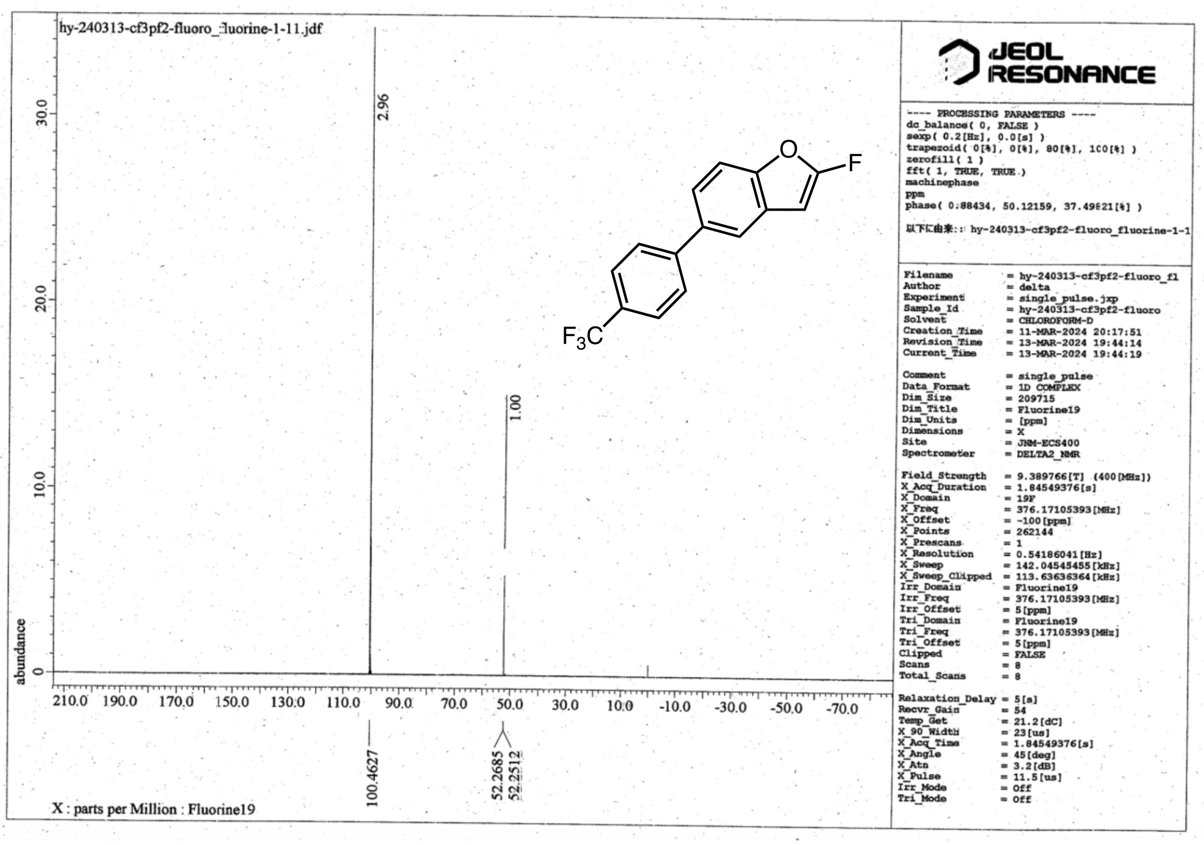
hy-240313-cf3pf2-fluoro_fluorine-1-11.jdf
2.96
1.00
F
O
F3C
abundance
X : parts per Million : Fluorine19
100.4627
52.2685
52.2512
JEOL RESONANCE
---- PROCESSING PARAMETERS ----
dc_balance(0, FALSE)
sexp(0.2[Hz], 0.0[s])
trapezoid(0[%], 0[%], 80[%], 100[%])
zerofill(1)
fft(1, TRUE, TRUE)
machinephase
ppm
phase(0.88434, 50.12159, 37.49821[%])
以下に由来:: hy-240313-cf3pf2-fluoro_fluorine-1-1
Filename = hy-240313-cf3pf2-fluoro_fl
Author = delta
Experiment = single_pulse.jxp
Sample_Id = hy-240313-cf3pf2-fluoro
Solvent = CHLOROFORM-D
Creation_Time = 11-MAR-2024 20:17:51
Revision_Time = 13-MAR-2024 19:44:14
Current_Time = 13-MAR-2024 19:44:19
Comment = single_pulse
Data_Format = 1D COMPLEX
Dim_Size = 209715
Dim_Title = Fluorine19
Dim_Units = [ppm]
Dimensions = X
Site = JNM-ECS400
Spectrometer = DELTA2_NMR
Field_Strength = 9.389766[T] (400[MHz])
X_Acq_Duration = 1.84549376[s]
X_Domain = 19F
X_Freq = 376.17105393[MHz]
X_Offset = -100[ppm]
X_Points = 262144
X_Prescans = 1
X_Resolution = 0.54186041[Hz]
X_Sweep = 142.04545455[kHz]
X_Sweep_Clipped = 113.63636364[kHz]
Irr_Domain = Fluorine19
Irr_Freq = 376.17105393[MHz]
Irr_Offset = 5[ppm]
Tri_Domain = Fluorine19
Tri_Freq = 376.17105393[MHz]
Tri_Offset = 5[ppm]
Clipped = FALSE
Scans = 8
Total_Scans = 8
Relaxation_Delay = 5[s]
Recvr_Gain = 54
Temp_Get = 21.2[dC]
X_90_Width = 23[us]
X_Acq_Time = 1.84549376[s]
X_Angle = 45[deg]
X_Atn = 3.2[dB]
X_Pulse = 11.5[us]
Irr_Mode = Off
Tri_Mode = Off

2-Phenyl-5-[4-(trifluoromethyl)phenyl]benzofuran (3fa)

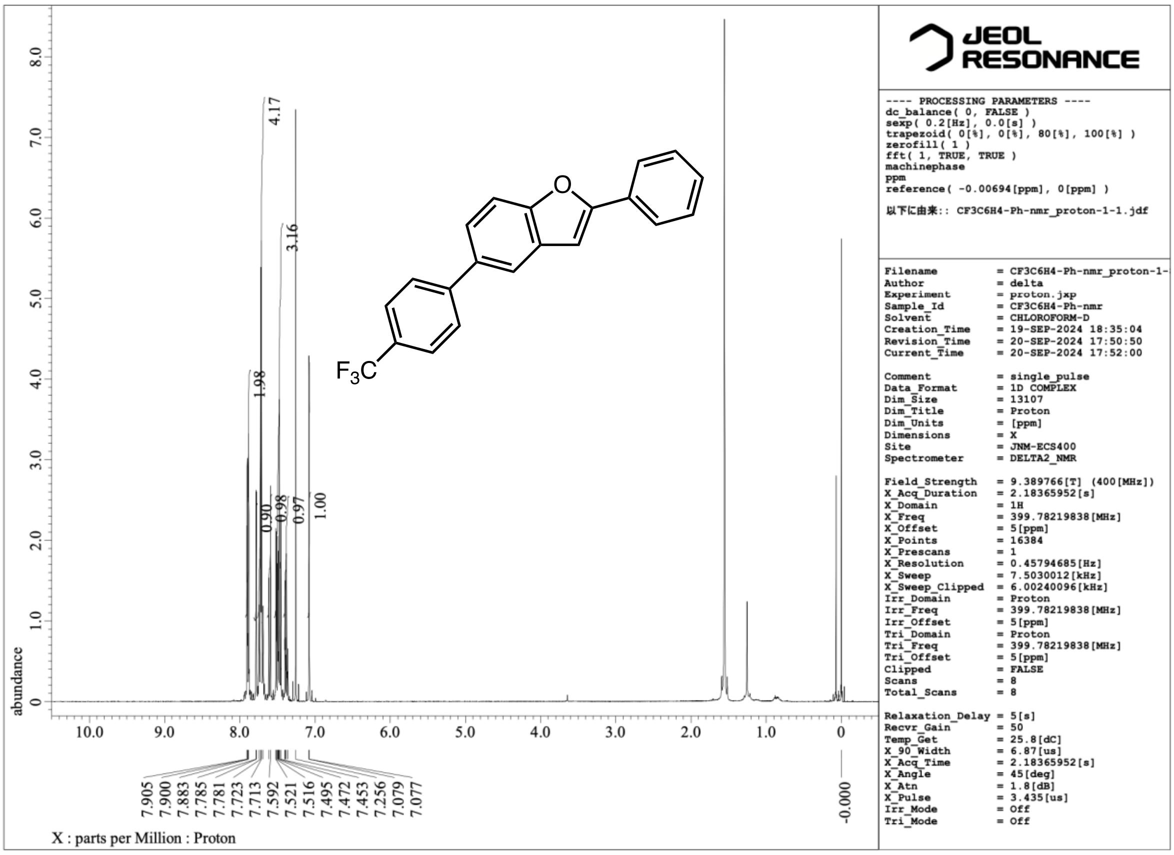

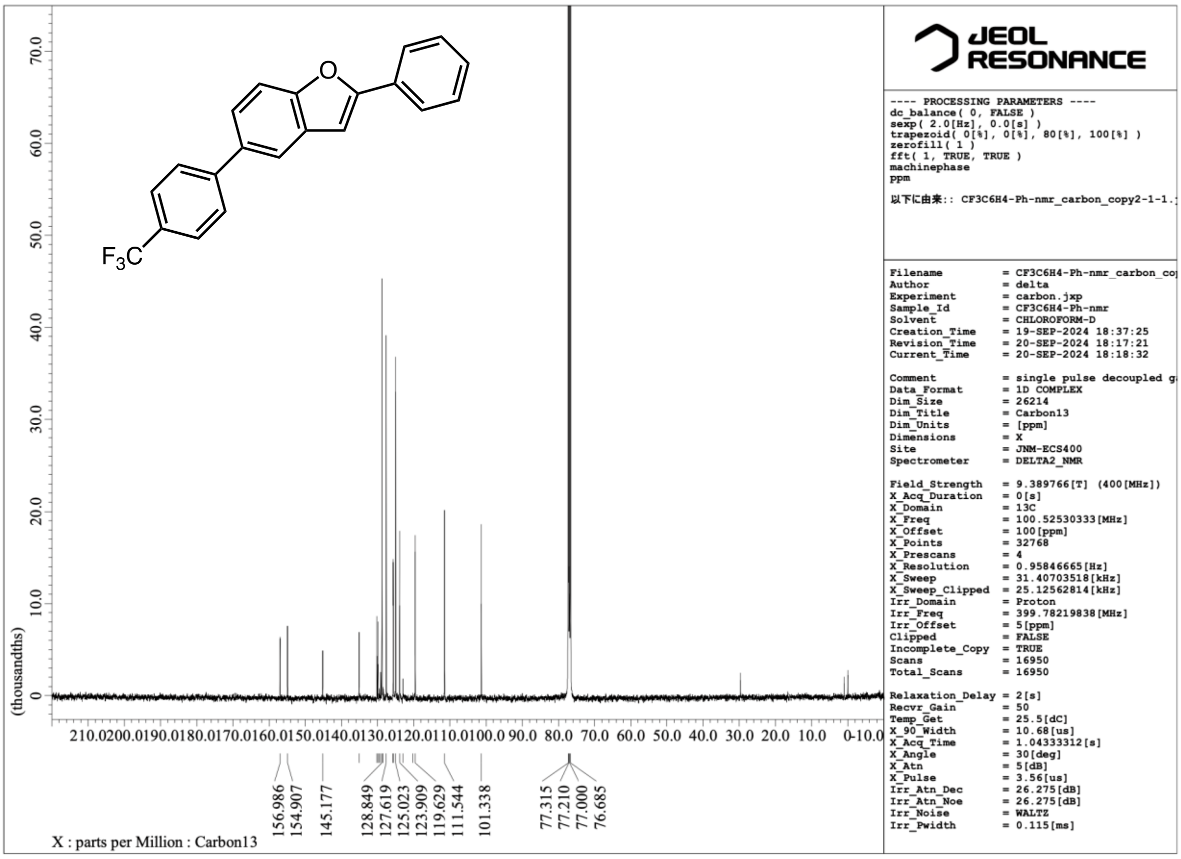

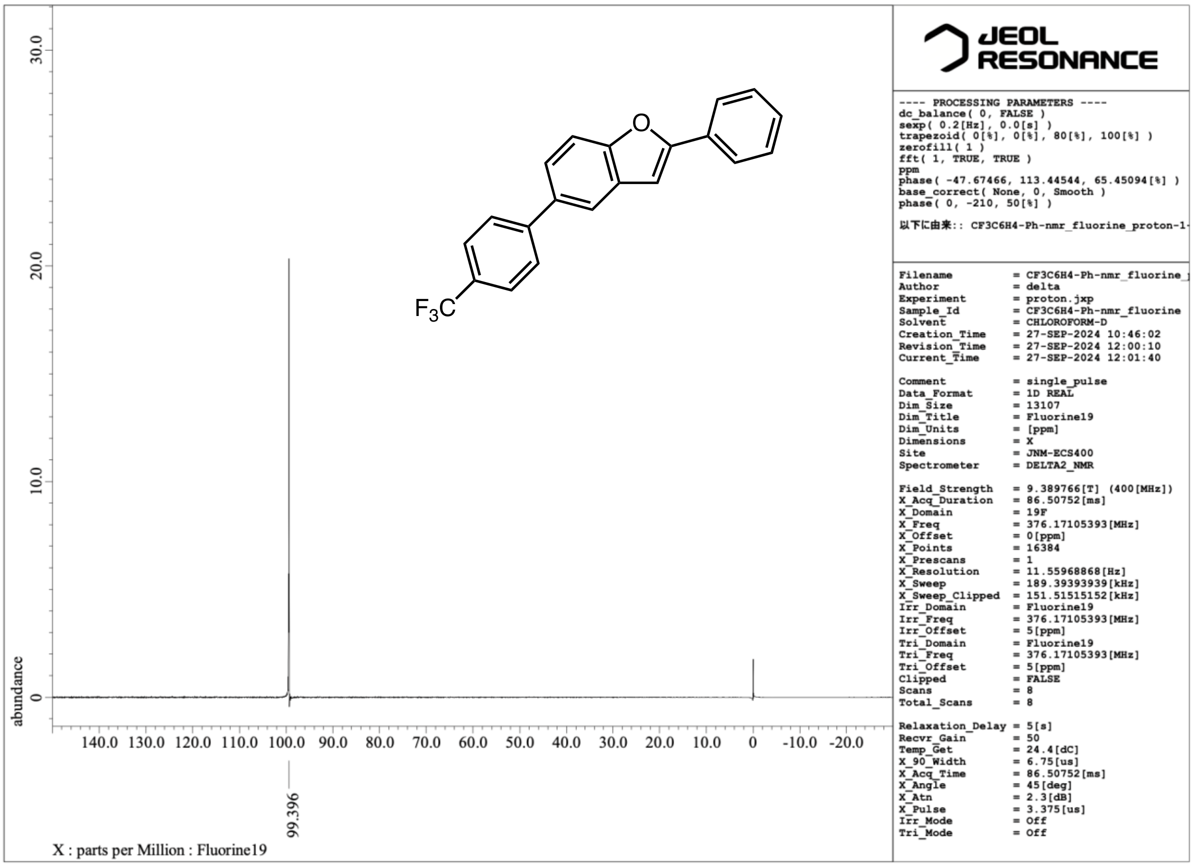
JEOL RESONANCE
---- PROCESSING PARAMETERS ----
dc_balance(0, FALSE)
sexp(0.2[Hz], 0.0[s])
trapezoid(0[%], 0[%], 80[%], 100[%])
zerofill(1)
fft(1, TRUE, TRUE)
ppm
phase(-47.67466, 113.44544, 65.45094[%])
base_correct(None, 0, Smooth)
phase(0, -210, 50[%])
以下に由来:: CF3C6H4-Ph-nmr_fluorine_proton-1-
Filename = CF3C6H4-Ph-nmr_fluorine_
Author = delta
Experiment = proton.jxp
Sample_Id = CF3C6H4-Ph-nmr_fluorine
Solvent = CHLOROFORM-D
Creation_Time = 27-SEP-2024 10:46:02
Revision_Time = 27-SEP-2024 12:00:10
Current_Time = 27-SEP-2024 12:01:40
Comment = single_pulse
Data_Format = 1D REAL
Dim_Size = 13107
Dim_Title = Fluorine19
Dim_Units = [ppm]
Dimensions = X
Site = JNM-ECS400
Spectrometer = DELTA2_NMR
Field_Strength = 9.389766[T] (400[MHz])
X_Acq_Duration = 86.50752[ms]
X_Domain = 19F
X_Freq = 376.17105393[MHz]
X_Offset = 0[ppm]
X_Points = 16384
X_Prescans = 1
X_Resolution = 11.55968868[Hz]
X_Sweep = 189.39393939[kHz]
X_Sweep_Clipped = 151.51515152[kHz]
Irr_Domain = Fluorine19
Irr_Freq = 376.17105393[MHz]
Irr_Offset = 5[ppm]
Tri_Domain = Fluorine19
Tri_Freq = 376.17105393[MHz]
Tri_Offset = 5[ppm]
Clipped = FALSE
Scans = 8
Total_Scans = 8
Relaxation_Delay = 5[s]
Recvr_Gain = 50
Temp_Get = 24.4[dC]
X_90_Width = 6.75[us]
X_Acq_Time = 86.50752[ms]
X_Angle = 45[deg]
X_Atn = 2.3[dB]
X_Pulse = 3.375[us]
Irr_Mode = Off
Tri_Mode = Off
F3C
O
abundance
30.0
20.0
10.0
0
140.0 130.0 120.0 110.0 100.0 90.0 80.0 70.0 60.0 50.0 40.0 30.0 20.0 10.0 0 -10.0 -20.0
99.396
X : parts per Million : Fluorine19

8. NMR and HRMS charts of nickelacyclopropane E_b

^{19}F NMR

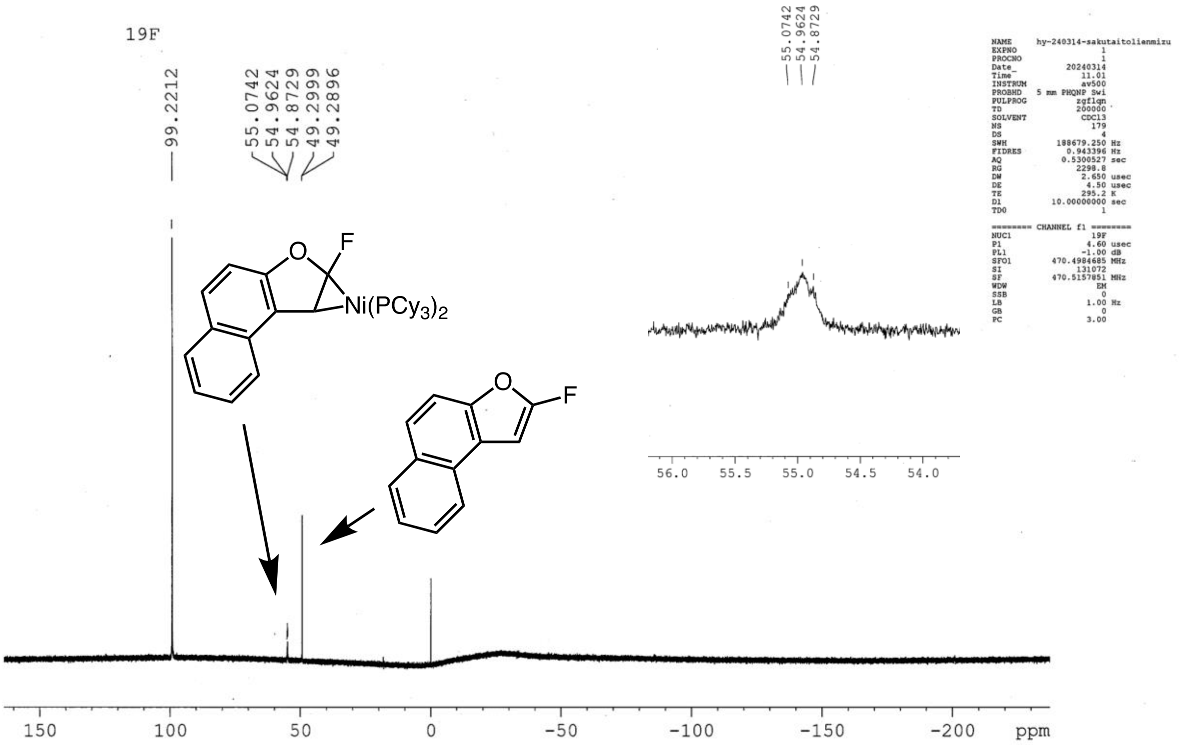

^{31}P NMR

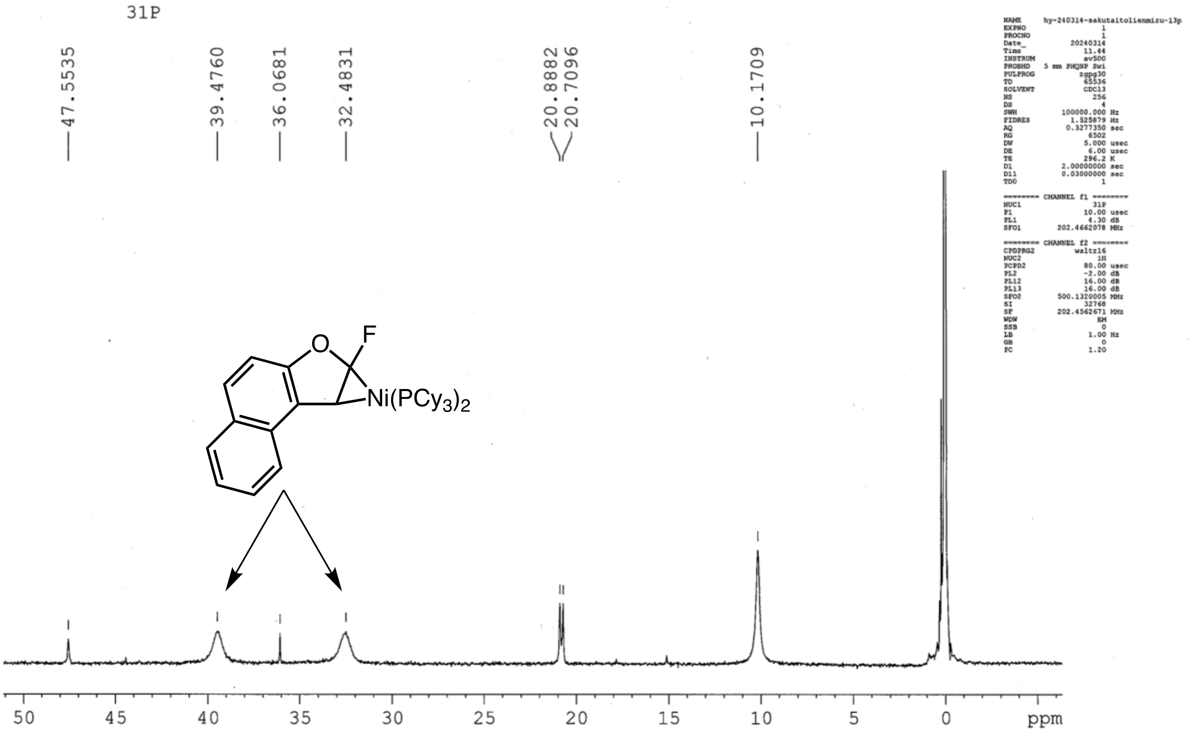

HRMS

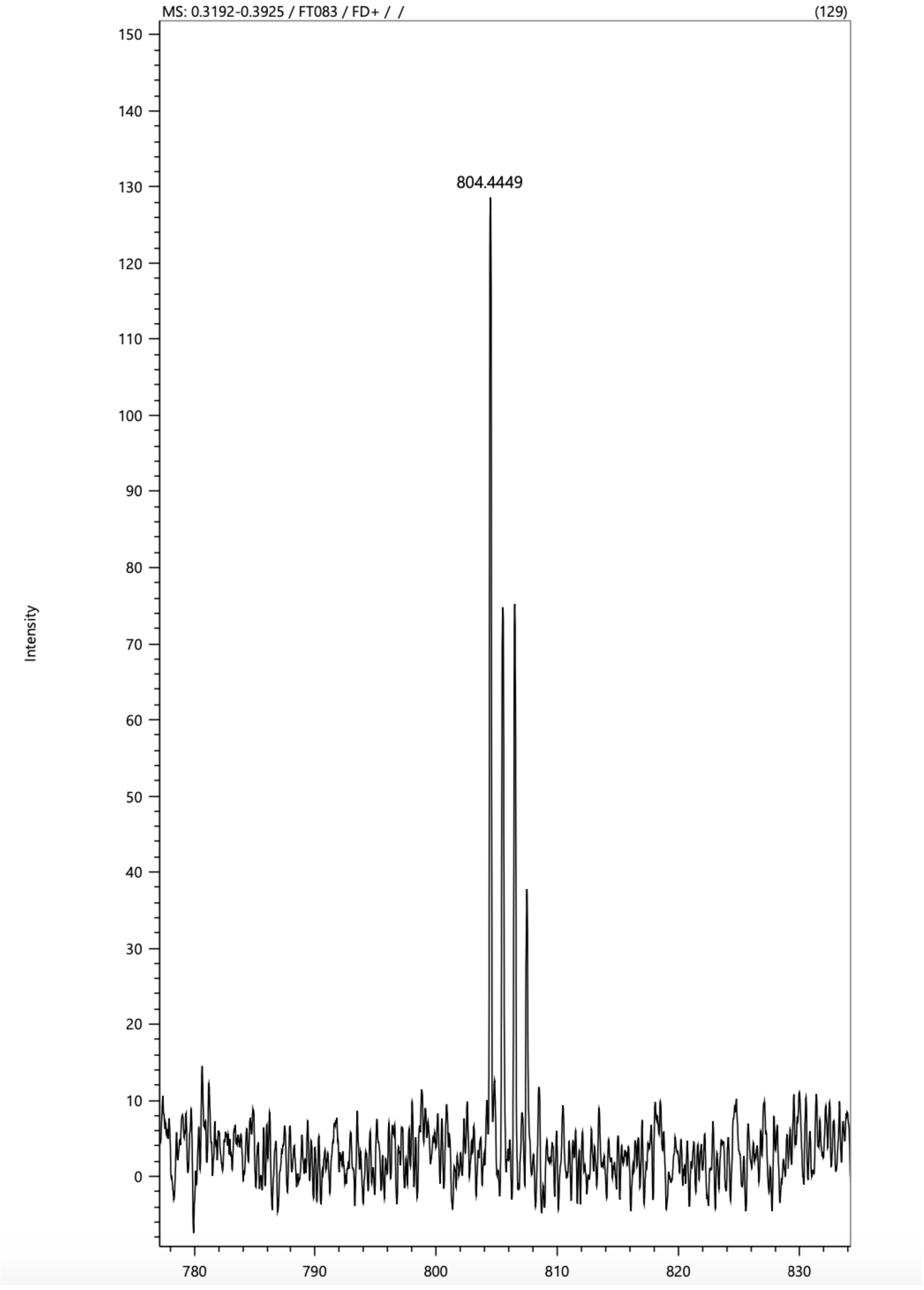
MS: 0.3192-0.3925 / FT083 / FD+ / /
(129)
804.4449
Intensity
150
140
130
120
110
100
90
80
70
60
50
40
30
20
10
0
780
790
800
810
820
830
